# Chronic lymphocytic leukemia patient-derived xenografts recapitulate clonal evolution to Richter transformation

**DOI:** 10.1038/s41375-023-02095-5

**Published:** 2023-11-28

**Authors:** Heribert Playa-Albinyana, Fabian Arenas, Romina Royo, Ariadna Giró, Irene López-Oreja, Marta Aymerich, Mònica López-Guerra, Gerard Frigola, Sílvia Beà, Julio Delgado, Pablo M. Garcia-Roves, Elías Campo, Ferran Nadeu, Dolors Colomer

**Affiliations:** 1 Experimental Therapeutics in Lymphoid Malignancies Group, Institut d’Investigacions Biomèdiques August Pi i Sunyer (IDIBAPS), Barcelona, Spain; 2 Centro de Investigación Biomédica en Red de Cáncer (CIBERONC), Instituto de Salud Carlos III, Barcelona, Spain; 3 University of Barcelona, Barcelona, Spain; 4 Barcelona Supercomputing Center (BSC), Barcelona, Spain; 5 Hematopathology Section, Pathology Department, Hospital Clínic, Barcelona, Spain; 6 Molecular Pathology of Lymphoid Neoplasms Group, Institut d’Investigacions Biomèdiques August Pi i Sunyer (IDIBAPS), Barcelona, Spain; 7 Hematology Department, Hospital Clínic, Barcelona, Spain; 8 Lymphoid Neoplasms Group, Institut d’Investigacions Biomèdiques August Pi i Sunyer (IDIBAPS), Barcelona, Spain; 9 Institut d’Investigació Biomèdica de Bellvitge (IDIBELL), L’Hospitalet del Llobregat, Barcelona, Spain

**Keywords:** Cancer models, Cancer genomics

## Abstract

Chronic lymphocytic leukemia (CLL) is a B-cell neoplasm with a heterogeneous clinical behavior. In 5–10% of patients the disease transforms into a diffuse large-B cell lymphoma known as Richter transformation (RT), which is associated with dismal prognosis. Here, we aimed to establish patient-derived xenograft (PDX) models to study the molecular features and evolution of CLL and RT. We generated two PDXs by injecting CLL (PDX12) and RT (PDX19) cells into immunocompromised NSG mice. Both PDXs were morphologically and phenotypically similar to RT. Whole-genome sequencing analysis at different time points of the PDX evolution revealed a genomic landscape similar to RT tumors from both patients and uncovered an unprecedented RT subclonal heterogeneity and clonal evolution during PDX generation. In PDX12, the transformed cells expanded from a very small subclone already present at the CLL stage. Transcriptomic analysis of PDXs showed a high oxidative phosphorylation (OXPHOS) and low B-cell receptor (BCR) signaling similar to the RT in the patients. IACS-010759, an OXPHOS inhibitor, reduced proliferation, and circumvented resistance to venetoclax. In summary, we have generated new RT-PDX models, one of them from CLL cells that mimicked the evolution of CLL to RT uncovering intrinsic features of RT cells of therapeutical value.

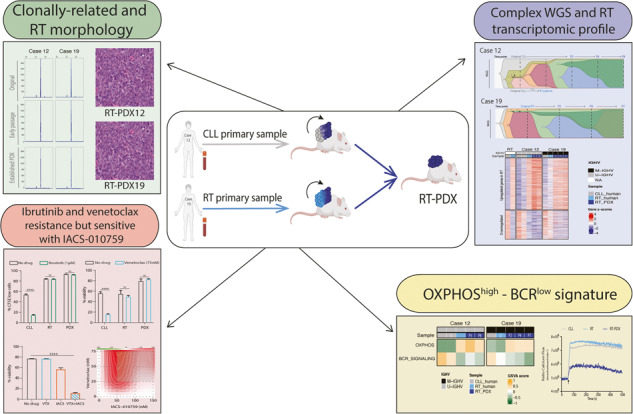

## Introduction

Chronic lymphocytic leukemia (CLL) is a complex and heterogeneous disease characterized by the proliferation and accumulation of CD5^+^ B cells in peripheral blood (PB), bone marrow (BM), and lymphoid tissues [1, 2]. Genetic and epigenetic mechanisms cooperate with microenvironmental factors to dictate clinical evolution [3–6]. CLL may have a long indolent and stable phase of the disease, but can also progress into a more aggressive disease with recurrent relapses or even transforming into a diffuse large-B cell lymphoma, a process known as Richter transformation (RT) [2, 7]. This transformation occurs in 5–10% of patients, mostly in those with relapsed or refractory disease to therapy. Patients with RT respond poorly to current therapies and have a dismal prognosis [8, 9]. RT usually has the histological characteristics of a diffuse large B-cell lymphoma (DLBCL), but with specific molecular lesions, such as specific stereotyped BCR immunoglobulins (subset #8), *NOTCH1* mutations, *TP53* inactivation, *MYC* translocations or amplifications, *CDKN2A* deletions, and complex karyotypes [7, 8]. Recently, new genetic, transcriptomic and epigenomic studies have described crucial pathways related to RT, such as DNA damage, MYC signaling, immune evasion, chromatin modification, cell cycle and PI3K signaling and oxidative phosphorylation (OXPHOS) [10–12].

Generation of adequate pre-clinical mouse models reflecting CLL/RT biology has not yet been successfully achieved [13]. The first and most used genetically engineered mouse model of CLL is the T-cell leukemia/lymphoma 1 transgenic (Eμ-TCL1) model that triggers the development of clonal CD5^+^ B cells in older mice, and develops an aggressive CLL variant [14, 15], lacking the recurrent driver gene mutations found in primary CLL [16]. Recently, new engineering strategies based on CRISPR-Cas9 editing have been described generating valuable tools for functional and treatment studies [16, 17]. Patient-derived xenograft (PDX) models, generated after injecting primary tumor cells into immunocompromised mice, have also emerged as a promising tool to track disease evolution and to test new treatment options [18, 19], although few PDX have been described in CLL, and all of them were generated from RT samples [20–22].

Here we describe the generation of two new RT-PDX models, one of them originated from a CLL sample mimicking the transformation of CLL to RT clinically observed in the follow-up of the patient. Both RT-PDXs maintain the clonal relationship, similar genomic and transcriptomic profiles compared to the RT tumors observed in the patients, but with a high subclonal heterogeneity and clonal evolution during PDX generation. These RT-PDXs were insensitive to BTK and BCL2 inhibitors as observed in primary RT cells. We propose that OXPHOS inhibition might be a novel therapeutic option for RT patients showing a synergistic effect with venetoclax. PDXs in CLL might recapitulate the biology of the tumors providing a new tool to study the pathogenesis of the disease and to assess the efficacy of new therapies.

## Methods

### Primary samples and cell lines

Cryopreserved peripheral blood mononuclear cells (PBMCs) (≥90% tumor CD19^+^/CD5^+^ B cells) from patients diagnosed with CLL and RT were obtained from the Hematopathology collection (Biobank Hospital Clínic-IDIBAPS; R121004-094). Informed consent was obtained according to the Institutional Review Board of the Hospital Clínic of Barcelona, which also approved the study. Clinical and biological characteristics of the patients are shown in Table S1. CLL MEC-1 cell line and the human bone-marrow derived stromal cell line HS-5 were cultured as described in supplemental Methods.

### Generation and characterization of PDX

A total of 2.5 × 10^7^ cells at a ratio of 40:1 B-cell:T-cell were resuspended in Matrigel TM Basement Membrane Matrix (BD Biosciences, Franklin Lakes, NY, USA) and sub-cutaneous (SC) injected in one flank of immunocompromised mice NOD-*scid* IL2rγ^null^ (NSG) mice (Charles Rivers Laboratories, Wilmington, MA, USA) and left to engraft. When tumor volume reached ≥1.5 cm^3^, mice were euthanized and tumor masses were collected, disrupted, and reinjected as a single-cell suspension with Matrigel in new NSG mice. For the intravenous (IV) model, 10^7^ cells from each PDX were resuspended in media and injected in the tail of NSG mice and engraftment was monitored. Additional details about PDX generation, their phenotype, histology, characterization and in vivo response to drugs are found in supplemental Methods.

### Whole-genome sequencing and RNA-seq analyses

Purified human DNA from PDX samples was subjected to whole-genome sequencing (WGS) using the TruSeq DNA PCR-Free library preparation and sequenced on a NovaSeq 6000 (Illumina, San Diego, CA, USA; 2 × 150 bp, mean coverage 30x). RNA-seq was performed on purified RNA samples using the Stranded mRNA Prep Ligation kit and sequenced on a NextSeq 2000 (Illumina; 2 × 50 bp, aiming at >40 million reads/sample). All bioinformatic analyses were performed as previously described [11] and are detailed in supplemental Methods.

### Functional studies

In vitro studies, fluorescence in situ hybridization (FISH), western blot experiments, proliferation and cytotoxicity assays, calcium flux analysis, and oxygen consumption were performed as detailed in supplemental Methods.

### Additional statistical analyses

Statistical data analysis for single comparison between control and treatment samples were performed in Prism Software 8.0 (GraphPad Software, New York City, NY, USA) based on Student *t-test* (two-tailed). For multiple comparisons one-way ANOVA with Tukey’s test was done. Drug interaction for synergy were performed on a ZIP [23] model in SynergyFinder [24] No samples or animals were excluded from the analyses. Randomization by age and weight was done in animal studies. The statistical test used for each data set is indicated in the figure legends.

## Results

### Establishment of PDX models from CLL/RT primary samples

**Fig. 1:**
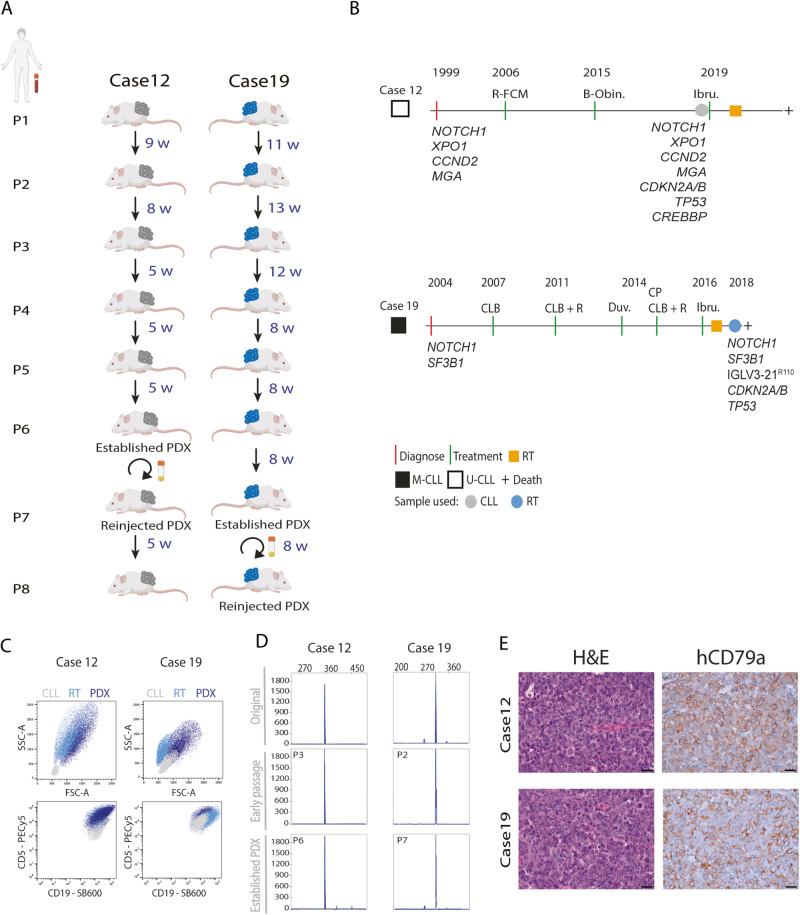
Description and characterization of PDX models. **A** Schematic representation of PDX generation from cases 19 (RT, cells in blue) and 12 (CLL, cells in gray). In dark blue is depicted the weeks (w) between passages. The circular arrow indicates that the cryopreserved PDX cells were reinjected and regrown. Cryopreserved established PDXs were able to engraft after eight weeks for case 19 and after five weeks for case 12, similar to defined time for PDX establishment. **B** Clinical evolution and main mutations detected in case 12 (white square) and case 19 (black square). Gray (CLL) and blue (RT) dots depict the time-point when cells were collected for PDX generation. Orange square indicates the time of RT diagnosis. Abbreviations: B-Obin bendamustine + obinutuzumab; CLB clorambucil, CLL chronic lymphocytic leukemia, Duv duvelisib, CP cyclophosphamide, Ibru ibrutinib, M-CLL immunoglobulin heavy chain mutated CLL, R rituximab, R-FCM rituximab + cyclophosphamide + fludarabine + mitoxantrone, RT Richter transformation, U-CLL, immunoglobulin heavy chain unmutated CLL. **C** Flow cytometry analyses of primary samples (CLL [gray] and RT [light blue]) and PDXs (dark blue) from cases 12 and 19. Forward scatter (FSC) vs. side scatter (SSC) plot for cell size and CD19 (SuperBright600) vs. CD5 (PE-Cy5) plot for tumor cell identification. **D** Detection of clonal rearrangement by PCR of immunoglobulin heavy chain (IGH) FR1 region analyzed by GeneScan in purified (hCD19^+^) cells. Original: DNA from patient sample used for PDX generation; Early passage: third PDX12 passage (PDX12-P3) and second PDX19 passage (PDX19-P2); Established PDX: sixth PDX12 passage (PDX12-P6) and seventh PDX19 passage (PDX19-P7). All PDX samples were clonally related as presented the same peak, identically to the original counterpart. **E** Morphology of sections from PDX12 and PDX19 tumor masses stained with hematoxylin/eosin (H&E) and human CD79a. All images were acquired at ×60 magnification.

Twenty millions of tumoral B cells and autologous activated T cells (ratio 40:1) from 11 CLL/RT patients (Table S1) were injected SC into NSG mice and let to engraft until tumor masses were palpable ( ≥ 1.5cm^3^). Engraftment was achieved in six cases, being clonally related to the original CLL/RT in case 12 (after 9 weeks) and in case 19 (after 11 weeks) and nonrelated in four cases (17, 27, 145, and 1228). Palpable masses were not detected after 40 weeks in the remaining five cases (Fig. S1). Stabilized PDX models took 32 weeks for case 12 (PDX12) and 60 weeks for case 19 (PDX19) (Fig. 1A).

PDX12 was generated from CLL PBMCs collected at relapse after bendamustine plus obinotuzumumab treatment, and before ibrutinib. This patient developed a RT five months after treatment with ibrutinib. PDX19 was generated from PBMCs from a patient with a clonally–related RT. This patient had been diagnosed with CLL 14 years before RT and received multiple lines of treatment including chlorambucil (CLB), CLB and rituximab, duvelisib, cyclophosphamide and prednisone, and ibrutinib (Fig. 1B). The diagnosis of RT was established in peripheral blood samples of the two patients and additionally in the bone marrow of case RT12. All these samples showed between 20-50% of large, very atypical B-cells with centroblastic and immunoblastic morphology (Fig S2A). Flow cytometry also detected a population of 25-70% large cells in all the samples.

Flow cytometry analysis of both PDXs showed that the cells were larger and more complex than the CLL cells, and similar to the RT of the patients. These cells also expressed the CLL markers, CD19 and CD5 (Fig. 1C; Fig. S2B). Analysis of the immunoglobulin heavy chain gene (IGHV) rearrangement confirmed the clonal relationship between the original CLL/RT and PDXs (Fig. 1D). Histological and immunohistochemical (IHC) analysis of these tumors showed a diffuse infiltration of large human CD79a-positive B cells (Fig. 1E). Few residual T cells were detected by flow cytometry (Fig. S3).

**Fig. 2:**
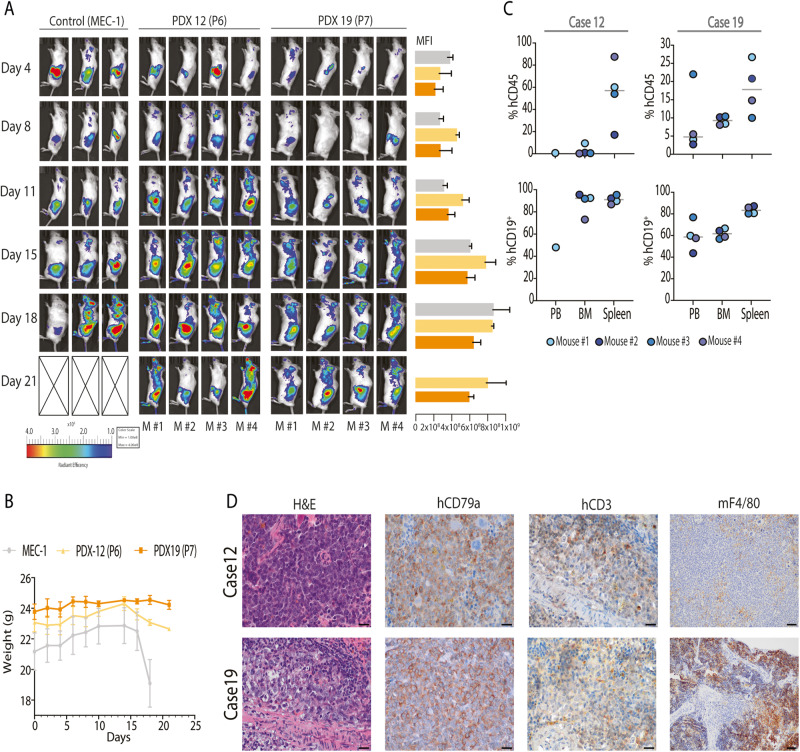
Generation of an in vivo RT model. **A** Tumor infiltration was tracked using IVIS® imager with fluorescent probe XenoLight 2-DG-750 every 3-4 days in NSG mice (11 cases): control (MEC-1 cell line) (*n* = 3), PDX12 (P6) (*n* = 4) and PDX19 (P7) (*n* = 4). Median Fluorescence Intensity (MFI) is depicted in a bar chart as a mean of each group: MEC-1 control (light gray), PDX12 (P6) (light orange) and PDX19 (P7) (dark orange). **B** Mice weight evolution from PDX12 (light orange), PDX19 (dark orange) and MEC-1 control (light gray). Data is presented as mean values ± SD. **C** Organ infiltration was determined by flow cytometry in peripheral blood (PB), bone marrow (BM) and spleen. First, hCD45 population was identified [top] and after gating the population, tumor cells (CD19^+^ CD5^+/-^) were selected [bottom]. Tumor B-cell infiltration in PB was 41.3% (only one mouse) for PDX12 and 59.4 ± 13.6% [PDX19]; in BM was 88.2 ± 10.2% [PDX12] and 61.6 ± 4.5% [PDX19], and in the spleen was 91.0 ± 3.6% [PDX12] and 83.5 ± 3.5% [PDX19]. Data from Control (MEC-1) is not shown. Data is presented as mean values ± SD. **D** Sections from NSG spleens from PDX12 and PDX19 stained with H&E, human CD79a, human CD3 and mouse F4/80. All images were acquired at ×60 magnification except mF4/80 images acquired at ×20 magnification.

To analyze the tumorigenic capacity of these PDXs, we injected 10^7^ PDX cells IV in NSG mice and let them spread. MEC-1 cell line was used as a reference for engraftment [25]. In the PDX injected mice, infiltration was detected at the left upper quadrant of the abdomen (spleen) and in some mice also at back limbs (Fig. 2A). Mice were sacrificed after 21 days of inoculation in the PDX12 and PDX19 groups and after 18 days in the MEC-1 group due to loss of weight (Fig. 2B). Spleen, PB and BM were analyzed, the spleen being the most infiltrated organ (54.7 ± 10.2% hCD45^+^ in PDX12 and 18.1 ± 7.2% hCD45^+^ in PDX19) (Fig. 2C). IHC analysis showed a diffuse infiltration of the spleen by human large CD79a-positive B cells, as well as murine macrophages (mF4/80) (Fig. 2D).

### Genomic characterization of PDX models

We analyzed by WGS the original CLL sample (CLL12), the RT patient’s sample (RT12) and 3 PDXs passages from PDX12 (PDX12-P3, PDX12-P6 and PDX12-P8) as well as the original RT sample (RT19) and 3 PDXs passages from PDX19 (PDX19-P2, PDX19-P4 and PDX19-P7) (Table S3). PDX12 and PDX19 genomes carried a median of 2.5 and 2.8 mutations per megabase respectively, similar to the original samples (2.5 for CLL12, 2.7 for RT19) (Tables S5 and S6). The number of copy number alterations (CNAs) and structural variants (SVs) in PDX12 (24 CNAs and 35 SVs) was similar to the original CLL sample (18 CNAs and 31 SVs), whereas it increased in PDX19 (54 CNAs and 150 SVs) compared to the parental RT19 (25 CNAs and 117 SVs) (Fig. S4; Tables S7, S8).

**Fig. 3:**
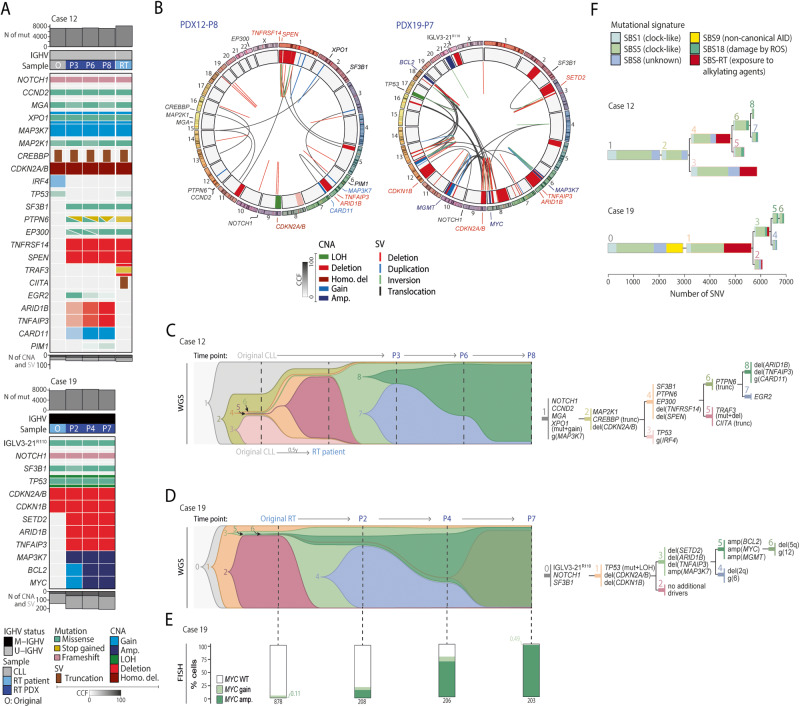
Genomic profile and clonal evolution in PDX. **A** Oncoprint showing driver alterations found in PDXs (dark blue) and/or CLL/RT original samples (gray and light blue, respectively). Each column represents a sample and genes are depicted in rows. The total number of mutations are shown at top, and the number of copy number alterations (CNAs) and structural variants (SVs) at bottom. The color transparency of the mutations and CNAs indicates the cancer cell fraction (CCF). CNAs and mutations are colored by type. O: original cells used for PDX generation; RT: original Richter transformation’s sample; P2 PDX passage 2, P3 PDX passage 3, P4 PDX passage 4, P6 PDX passage 6, P7 PDX passage 7, P8 PDX passage 8. IGHV status, type of sample and type of mutation and CNA are colored by type. **B** Circos plots from last passage from case 12 (PDX12-P8) and case 19 (PDX19-P7). Plots displays the SVs (links) and CNAs (inner circle). Driver genes altered are annotated. Chromosomes are depicted in the outer circle. CNAs and SV are colored by type. **C** Clonal evolution along the PDX generation inferred from whole-genome sequencing (WGS) in case 12. Each subclone is depicted by a different color and number. The height of each subclone in each time point (vertical dashed line) is proportional to their CCF. The phylogeny of the subclones with the main driver alterations is shown [right]. **D** Clonal evolution along the PDX generation inferred from WGS data in case 19. Data is represented as described in panel **C**. **E** Percentage of *MYC* gains (light green) or amplifications (dark green) from case 19 in each time-point determined by FISH. The total number of cells per sample is shown at the bottom of the bar chart. The number of cells for each subgroup is detailed in Table S10. **F** Phylogenetic reconstruction of subclones and contribution of the mutational signatures to their mutational profile. Each mutational signature is colored by type.

In case 12, the original CLL sample (CLL12) carried alterations in *NOTCH1*, *CCND2, MGA*, *XPO1*, *MAP3K7*, *MA2K1*, *CREBBP* and *CDKN2A/B*. PDX samples carried these alterations together with aberrations in *SF3B1*, *PTPN6*, *EP300*, *TNFRSF14* and *SPEN*, which were also observed in the patient’s RT sample (RT12). Nonetheless, two alterations affecting *CIITA* and *TRAF3* were observed in RT12 but not in the PDX, suggesting that a slightly different RT subclone expanded in mice. In PDX12, new alterations affecting *EGR2, ARID1B, TNFAIP3, CARD11*, and *PIM1* were detected at different clonalities along the passages suggesting an ongoing clonal evolution (Fig. 3A). Simple and complex chromosomal alterations affecting driver genes were observed in PDX12; a genomic profile that was comparable to RT12 but not to CLL12 (Fig. 3B; Fig. S4B). Clonal analysis revealed that the subclones expanding in RT12 (subclone #5) and in PDX12 (subclone #6) were present in a small fraction of cells in CLL12 (Fig. 3C). While subclone #5 carried the deletion of *TRAF3* mediated by an aberrant class-switch recombination (CSR) and a point mutation in the V(D)J IGHV gene rearrangement, subclone #6 had *PTPN6* truncated by an aberrant CSR and two-point mutations in the VJ gene rearrangement of the light chain, highlighting convergent evolution in descendent RT subclones (Fig. S5). In PDX12-P3, the major subpopulation was subclone #7, which originated from subclone #6 acquiring a mutation in *EGR2*. Contrarily, subclone #8, which also originated from subclone #6, acquired alterations in *ARID1B*, *TNFAIP3* and *CARD11*, and expanded along the passages overcoming subclone #7 in PDX12-P6 and PDX12-P8. Of note, subclones #7 and #8 converged into the acquisition of alterations in NF-kB pathway (Fig. 3C, D).

In case 19, the original RT19 carried mutations in IGLV3-21^R110^, *NOTCH1*, *SF3B1* and *TP53* as well as deletions of *CDKN2A/B* and *CDKN1B*. PDX samples maintained these alterations and acquired deletions of *SETD2*, *ARID1B* and *TNFAIP3*, and amplifications of *MAP3K7*, *BCL2* and *MYC* (Fig. 3A). We observed that *BCL2* and *MYC* amplifications increased over the passages suggesting an ongoing clonal evolution (Fig. S6A). We validated the overexpression of *MYC*, *BCL2* and *MAP3K7* by western blot and RNA-seq (Fig. S6B, C). Several chromothriptic events were detected in PDX19 involving one or multiple regions, such as 8q, 10q and 18q, and leading to amplifications and deletions (Fig. 3B). The reconstruction of the subclonal architecture and its dynamics uncovered that the main subclone in RT19 (subclone #2) was not engrafted in mice (Fig. 3D). Contrarily, the engrafted subclones were derived from a small subclone present in the RT19 that carried alterations in *SETD2*, *ARID1B, TNFAIP3* and *MAPK37* (subclone #3). Subclones #2 and #3 originated from subclone #1, which harbored the RT driver alterations *TP53*, *CDNKN2A/B* and *CDKN1B*. Although the subclone that dominated the initial passage (PDX19-P2) was subclone #4, originated from subclone #3, which harbored deletions in 2q and gains in chromosome 6, this subclone diminished in the subsequent passages. Contrarily, subclone #5, which originated from subclone #3 and acquired alterations in *BCL2* and *MYC*, was the dominant population at PDX19-P4. Intriguingly, subclone #5 was predicted to be present in a small fraction of cells in the original sample. Indeed, we found 0.11% of cells carrying *MYC* amplification in the original RT sample by FISH, which expanded to 14.9%, 67.5%, and 98.5% of cells in the subsequent passages (Fig. 3E; Fig. S6D and Table S10). In the last passage (PDX19-P7), subclone #6, which originated from subclone #5 and carried a deletion in 5q and gains in chromosome 12, represented the major tumor subpopulation.

To understand the mutational processes operating in the distinct subclones, we measured the contribution of the mutational processes previously identified in CLL and RT [11] in each subclone (Fig. 3F, Table S11). We did not observe differences in the mutational signatures found in subclones expanding in patients and mice, suggesting that expanding subclones in PDX models does not lead to a distinct mutational profile. Among the mutational signatures identified, the single base substitution (SBS) signature 18 (SBS18), which has been associated to damage by reactive oxygen species (ROS), was found in advanced RT subclones present in the patient and mice in both cases. The recently described SBS-RT associated to cell exposure to the alkylating agents bendamustine and chlorambucil [11] was found in the subclones from case 19 expanding in the patient (subclone #2) and in mice (#3), thus uncovering that these two subclones diverged from the parental subclone #1 early in the CLL phase of the disease when the patient received chlorambucil.

### PDX cells exhibit a RT transcriptional profile

**Fig. 4:**
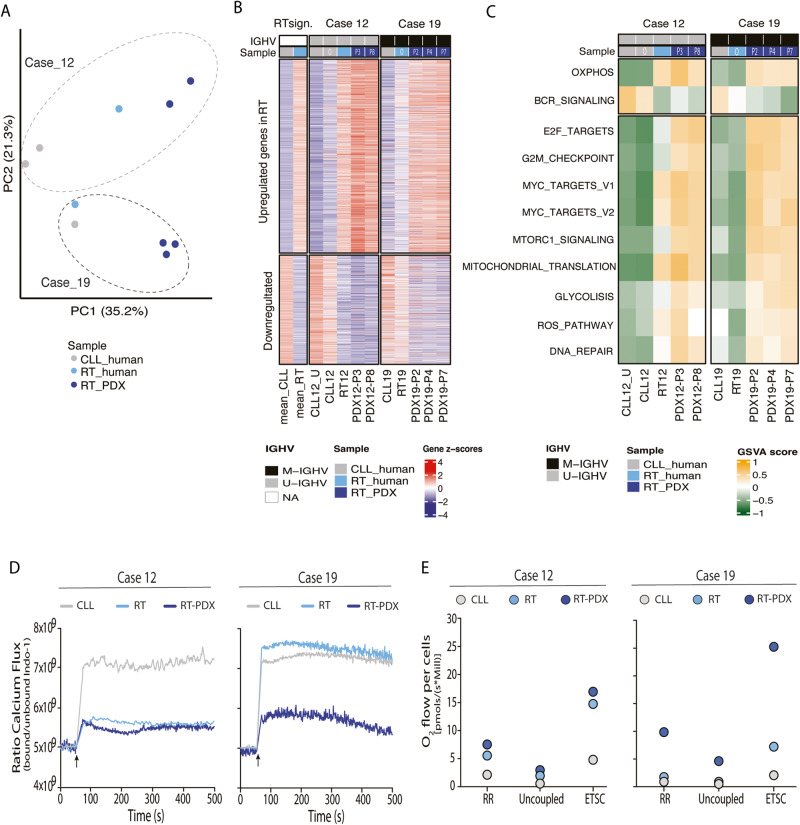
PDXs have a transcriptomic profile of RT and showed an OXPHOS^high^–BCR^low^ phenotype. **A** Principal components analysis based son RNA-seq data from original CLL/RT and PDX samples from both cases. **B** Heatmap showing the 2,244 differentially expressed genes (DEG) between RT and CLL according to Nadeu et al. [11] in samples from cases 12 and 19. RT sign: signature described in Nadeu et al.^12^ Case 12: a CLL sample collected prior any therapy (CLL12_U), the CLL sample used to develop the PDX (CLL12), the RT sample (RT12) and two PDXs (PDX12-P3 and PDX12-P8); for case 19: CLL19 (CLL sample), the original RT sample (RT19) and the three PDXs (PDX19-P2, PDX19-P4 and PDX19-P7); NA: not applicable. **C** Heatmap showing gene set variation analysis (GSVA) score of gene sets modulated in RT according to Nadeu et al.^12^ in samples from cases 12 and 19. **D** Calcium kinetics of tumor cells (CD19^+^ CD5^+^) from original CLL/RT and PDX from case 12 and 19. Basal calcium was adjusted at 5×10^9^ Indo-1 ratio (bound/unbound) for 60 seconds prior cell stimulation with F(ab’)2 anti-human IgM + H_2_O_2_ at 37 °C and 4-hydroxytamoxifen (4-OHT). Then, Ca^2+^ flux was recorded up to 500 seconds. Black arrow indicates the time cells were stimulated. **E** Oxygen (O_2_) flux from original CLL/RT and PDX cells from case 12 and 19 at routine respiration (RR), oligomycin-inhibited leak respiration (Uncoupled), and after exogenous uncoupler stimulation. (ETSC). CLL samples are represented in gray, RT samples in light blue and PDX samples in dark blue.

We analyzed the transcriptional profile of CLL, RT and PDXs by RNA-seq (Table S12). An unsupervised principal component analysis showed that principal component 1 distinguished CLL vs RT and PDX samples, whereas the second component separates the two cases (Fig. 4A). The similarity between PDXs and RTs was also observed in the expression pattern of 2244 differentially expressed genes (DEGs) (1436 upregulated; 808 downregulated) previously identified in RT vs CLL analysis [11] (Fig. 4B). In case 12, although the PDX was generated from a CLL sample not exposed to BTK inhibition, it had a RT-like expression profile similar to the RT developed in the patient after treatment with ibrutinib. PDX19 showed a higher RT expression profile compared to the original RT sample (Fig. 4B).

According to our previous study [11], RT tumors showed an increased OXPHOS and decreased BCR signaling. We found that this OXPHOS^high^–BCR^low^ transcriptional axis was present in all PDXs samples (Fig. 4C). Additional, other pathways were upregulated in RT and PDXs (E2F, MYC and mTOR pathways; G2M checkpoints; mitochondrial translation; glycolysis; ROS; and DNA repair, among others) (Fig. 4C).

### BCR and OXPHOS activity in RT-PDXs

To validate the decrease of BCR activity in PDX samples we measured calcium flux upon BCR stimulation with anti-IgM. Cells from both PDXs presented lower calcium flux compared to CLL samples, indicative of lower BCR activity (Fig. 4D). RT cells from case 12 showed similar levels of BCR activity than PDX12, in contrast RT cells from case 19 showed calcium flux levels similar to CLL19, concordant with the presence of IGLV3-21^R110^ mutation that induces a constitutive BCR activation independent of external stimulus [26, 27] (Fig. S7).

We next analyzed mitochondrial respiration to validate the high OXPHOS signature observed by transcriptomic analysis. Cells from both PDXs had higher oxygen consumption rate (OCR) compared to CLL cells respiratory levels (routine respiration) (PDX12: 3.1-fold and PDX19: 10.9-fold) and at maximum electron transfer system capacity (ETSC) (PDX12: 3.5-fold and PDX19: 12.0-fold) (Fig. 4E). In case 12, the OCR from PDX and RT cells were similar, in contrast RT19 showed lower OCR than its PDX counterpart.

### PDX cells are resistant to ibrutinib and venetoclax

**Fig. 5:**
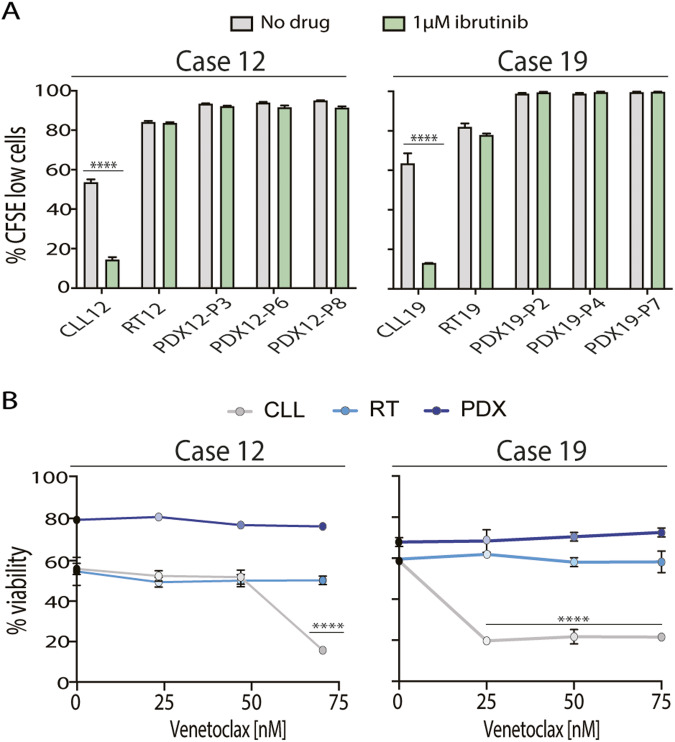
RTs and PDXs are resistant to ibrutinib and venetoclax treatment. **A** CLL/RT primary cells and PDX cells from cases 12 and 19 were cultured in enriched medium (EM) with ODN2006 + IL-15 and without (gray) or with (green) 1 µM ibrutinib for 6 days. Percentage of proliferating alive cells was determined by carboxyfluoresceinsuccinimidyl ester (CFSE) cell tracer. **B** Cells were treated with increasing doses of venetoclax (25, 50 or 75 nM) for 48 h and cell viability was determined by Annexin-V staining. Error bars: SD (*n* = 3). Statistical significance was considered when *P* value * <0.05; ** <0.01; *** <0.001 and **** <0.0001.

We tested the effect of ibrutinib on cell proliferation by incubating primary CLL and RT samples, and cells from PDXs for 6 days in an enriched medium containing ODN2006 + IL-15 (EM), which was the best condition culture to maintain viability and to induce proliferation on cells (Fig. S8). All cells proliferated under these conditions after 6 days, being proliferation of PDXs almost 100%. Incubation with ibrutinib did not affect cell proliferation neither in RT nor PDX cells but induced a significant reduction on proliferation in the two primary CLL samples collected prior ibrutinib treatment (CLL12 [73.0 ± 2.5%] and CLL19 [79.2 ± 1.5%]) (Fig. 5A; Table S16). Of note, although PDX12 was originated from an ibrutinib in vitro sensitive sample (CLL12), it was resistant to the drug (Fig. 5A).

Cells were also incubated with increasing doses of venetoclax (25, 50 and 75 nM) for 48 h in EM conditions (Table S17). All RT and PDX cells were resistant to venetoclax, while cells from CLL19 were sensitive to venetoclax at all doses tested (Fig. 5B), and CLL12 cells were only sensitive to venetoclax at high doses (75 nM) (Fig. 5B).

### OXPHOS inhibition as a new therapeutic target

**Fig. 6:**
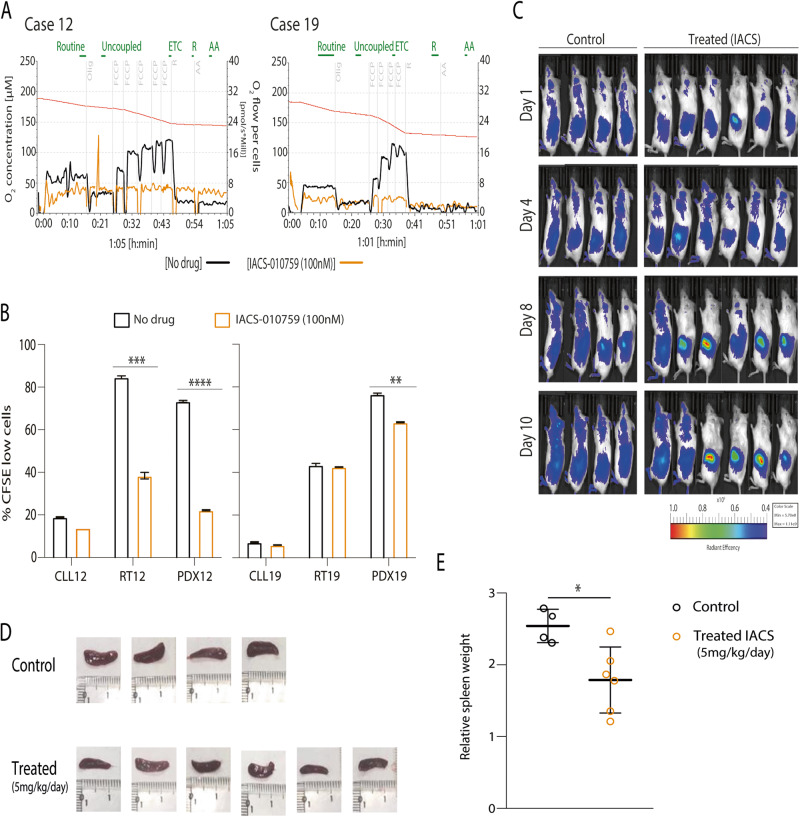
IACS-010759 inhibits proliferation on RT-PDX cells in vitro and in vivo. **A** PDX cells were treated with IACS-010759 for one-hour prior respirometry assays. Analysis of oxygen (O_2_) flow were done in samples without (green) or with 100 nM of IACS-010759 (orange). Initially, baseline cellular O_2_ is measured from basal respiration (routine). Next oligomycin (Olig.), a complex V inhibitor, is added analyzing the ATP-linked respiration and proton leak respiration (uncoupled). After, the protonophore carbonyl cyanide-p-trifluoromethoxyphenyl hydrazone (FCCP) is added to collapse the inner membrane gradient, allowing the electron transfer system capacity (ETC) to function at its maximal rate. FCCP is added until no more ETC is detected. Then, rotenone (R), inhibitor of complex I, and antimycin A (AA), inhibitor of complex III, are added to shut down ETC function, revealing the non-mitochondrial respiration. **B** Cell proliferation was analyzed in the original CLL/RT and PDX samples after 72 h without (green) or with 100 nM of IACS-010759 (orange). Percentage of proliferating cells was determined by CFSE cell tracer. Two technical replicates of original CLL/RT samples were performed and for PDXs samples, three technical replicates. Error bars: SD. Statistical significance was considered when *P* value * <0.05; ** <0.01; *** <0.001 and **** <0.0001. **C** In vivo effect of IACS-010759 in the RT-PDX12 NSG model. Ten NSG mice were used (4 untreated [Control] and 6 Treated [IACS]). Cells were injected IV and tumor infiltration was tracked with IVIS® imager using 100 nM of fluorescent probe XenoLight 2-DG-750. Whole body fluorescence of lateral decubitus was detected by imaging. Mice were sacrificed after 10 days of treatment (vehicle, *n* = 4) or IACS-010759 (*n* = 6) and spleens sizes **D** and weights **E** were analyzed. Spleens weights are relative to mice body from treated with vehicle (green) or IACS-010759 (orange). Statistical significance was considered when *P* value * <0.05.

As high OXPHOS levels were detected in both PDXs, we analyzed the biological effect of inhibiting OXPHOS activity with IACS-010759, a small molecule that targets complex I of the mitochondrial electron transport chain (ETC) [28], which is upregulated in our PDX samples (Fig. S9). First, we showed that 100 nM of IACS-010759, was able to inhibit ETC in both PDXs cells (Fig. 6A). The incubation of cells with IACS-010759 for 72 h induced a reduction on cell proliferation in all cases with high OXPHOS activity profile (54.6 ± 2.2% for RT12, 69.8 ± 0.5% for PDX12 and 17.2 ± 0.9% for PDX19) (Fig. 6B; Table S18). We studied the in vivo effect of IACS-010759 in these PDXs. Ten million cells from PDX12-P8 were injected IV, and after 7 days mice were treated orally with IACS-010759 (5 mg/kg once daily) for 10 days. Treated mice showed low tumor infiltration, and a reduction on spleen size and weight (Fig. 6C–E).

**Fig. 7:**
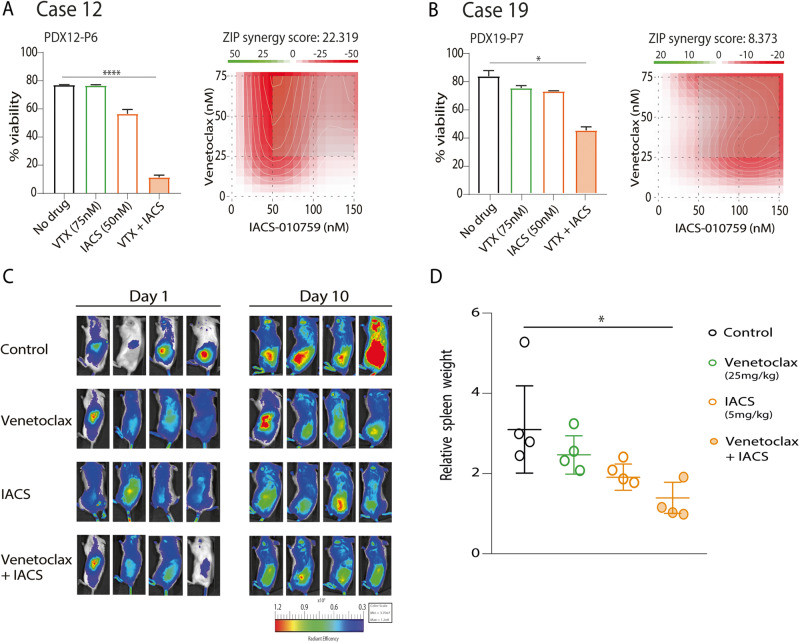
IACS-010759 circumvent venetoclax resistance in vitro and in vivo in RT and PDX cells. **A** Percentage of alive cells (Annexin V^-^) from PDX12, after 48 h of treatment with 75 nM of venetoclax (VTX) alone, 150 nM of IACS-010759 (IACS) alone and the combination of VTX + IACS. Drug interaction landscape and synergy score for the two drugs was calculated according to ZIP model. ZIP score 0–10 reflects an additive effect. **B** Percentage of alive cells (Annexin V^-^) from PDX19, after 48 h of treatment with 75 nM of VTX alone, 50 nM of IACS alone and the combination of VTX + IACS. Drug interaction landscape and synergy score for the two drugs was calculated according to ZIP model. ZIP score >10 reflects a synergistic effect. **C** In vivo effect of IACS-010759 5 mg/kg, venetoclax 25 mg/Kg and the combination in the RT-PDX12 NSG model. Tumor infiltration after one week of cell injection was tracked by optical in vivo imaging using fluorescent probe IRDye® 800CW 2-DG Optical Probe in NSG mice (4 per group). **D** Mice were sacrificed after 10 days of treatment and spleens sizes (*n* = 4) were analyzed. Statistical significance was considered when *P* value *<0.05; **<0.01; ***<0.001 and ****<0.0001 after one-way ANOVA statistical test. Error bars: SD (*n* = 3 or 4).

Since OXPHOS inhibition can overcome resistance to venetoclax [29, 30], we next analyzed the effect of combining IACS-010759 with venetoclax. PDX cells were incubated with different doses of IACS-010759 (50, 100 or 150 nM) and venetoclax (25, 50 or 75 nM) for 48 h, and the effect on the viability was analyzed (Table S19). PDX12 which was more resistant to venetoclax and presented a higher OXPHOS activity, showed a synergistic effect (ZIP score 22.319) at 50 nM IACS-010759 with 75 nM of venetoclax, with an 85.3 ± 2.2% of cell death similar to RT12 (ZIP score 33.439) (Fig. 7A, Fig. S10); whereas in PDX19, we observed an additive effect (ZIP score 8.373) using 150 nM IACS-010759 and 75 nM venetoclax, achieving a 46.2 ± 5.7% of cell death (Fig. 7B). These results were tested in vivo in our NSG-PDX model using venetoclax 25 mg/kg and IACS-010759 5 mg/kg once daily for 10 days. A modest effect of venetoclax was observed and the combination with IACS showed lower tumor infiltration than each drug alone and a reduction on spleen weight. (Fig. 7C, D).

We also tested if OXPHOS inhibition could overcome ibrutinib resistance. PDX cells were incubated with different doses of IACS-010759 and ibrutinib (1 or 5 µM) and did not show any additive or synergistic effect (Fig. S11; Table S20).

Recently, it has been described that CD40 stimulation increases OXPHOS and glycolysis in CLL cells, leading venetoclax resistance that can be counteracted by OXPHOS and mTOR inhibitors [31]. These pathways were upregulated in RT and PDXs (Fig. 4C). Therefore, we tested two different mTOR1 inhibitors (Rapamycin and Everolimus) and a glycolysis inhibitor (2-deoxyglucose [2-DG]) either alone or in combination with venetoclax. Neither mTOR inhibitors nor 2-DG were able to circumvent venetoclax resistance in PDX cells (Fig. S12).

## Discussion

PDX models are dynamic systems that recapitulate intertumoral and interpatient diversity, allowing the analysis of tumor evolution through serial propagation in mice [19]. In CLL, engraftment of primary cells into mice and the generation of PDX models have been a recurrent concern [13, 32, 33] with only few RT-PDX models reported [20, 34] being useful to explore novel therapies [34–36]. In this study, by co-xenotransplanting tumor B cells with autologous activated T cells into NSG mice [37], we have generated two new RT-PDXs clonally related to the original samples. PDX19 was derived from a blood sample diagnosed already with RT but surprisingly PDX12 was originated from a primary CLL sample, collected prior ibrutinib treatment. The patient also developed a RT five months after treatment. The result of this case demonstrates that RT cells were already present within the circulating CLL cells, confirming the concept of early seeding of RT subclones at early stages of the disease recently described [11, 12]. The diagnosis of the RT in these two patients was established in peripheral blood and bone marrow samples. Although RT is usually diagnosed in a tissue biopsy, the high number of large, very atypical and pleomorphic B-cells allowed the diagnosis of RT in these samples. The leukemic phase of RT is uncommon but has been recognized in the literature [38]. The development and progression of CLL is driven by a complex interaction between the tumor cells and cells from their microenvironment [39]. As our PDX cells have grown in immunodeficient NSG mice, the lack of a full tumor microenvironment [40] could be the responsible to trigger the expansion of these RT subclones. Recently, new CRISPR-Cas9 gene editing-engineered RT mouse model with selection of *TP53, MGA* and *CHD2* as CLL drivers have been described [41]. These RT mouse model could be useful to study the role of microenvironment in RT expansion.

WGS and RNA-seq analyses from sequential passages of each PDX allowed us to reconstruct the subclonal architecture, its dynamics, and transcriptomic profiles. These analyses uncovered a remarkable subclonal diversification of RT, which was missed in previous studies using patient samples [11, 12]. In addition, the detection of mutations associated with treatment regimens of the CLL phase in distinct RT subclones suggest that RT diversification might occur at early CLL stages. In our two PDXs, a small RT subclone present in the original sample engrafted in mice. This subclone generated two distinct subclones that co-occurred in the mice and compete during the generation of PDXs. In case 19, the engrafted subclone carried novel driver alterations, such as *MYC* and *BCL2* amplification [10, 41], compared to the RT counterpart. These alterations might probably explain the RT-like transcriptomic profile of this PDX compared to the more CLL-like profile [11] of the original RT sample. Our results also confirmed that *NOTCH1* is an important RT player, as both PDXs generated in this study and others [20, 36] carried this alteration. Our results also suggested that a compendium of CLL driver alterations such as *SF3B1*, alterations in cell cycle (*CDKN2A/B*), *MYC*, and NF-κB pathways could be acquired during the evolution of the disease. Recently, by modeling CLL transformation into RT through in vivo gene editing of only 6 loss-of-function driver genes, it has been reported that only *TP53*, the *MYC* negative regulator *MGA* and the chromatin remodeler *CHD2* promoted RT [42]. These genes were also altered in our models suggesting that these genetic events might facilitate the development of RT [43].

Our PDXs showed a consistent transcriptomic profile related to RT characterized by upregulation of cell proliferation, MYC targets, mTOR signaling, OXPHOS, mitochondrial translation, glycolysis, ROS, DNA damage pathways, and downregulation of BCR signaling [11]. The activation of OXPHOS pathway and downregulation of BCR signaling observed already at first passages of PDXs might explain their rapid expansion, since MYC-OXPHOS activity has been linked with a proliferative drive in CLL [42, 44, 45]. The low BCR activity in RT-PDXs and in human RT samples at transcriptional and functional level could explain the low response to BCR inhibitors [46, 47]. In contrast, studies of RT development in the TCL1 mice have shown that loss of *CDKN2A/B* together with IgM stimulation are needed [48], and are dependent of IgM stimulation for their survival [49]. Our results suggested that ibrutinib treatment is not the responsible of an OXPHOS^high^–BCR^low^ axis since RT-PDX12 was originated from a sample collected before ibrutinib treatment and mice were no treated with ibrutinib during PDX generation. The observation that PDX19 carrying an IGLV3-21^R110^ mutation, which confers a constitutive BCR activation [26, 27, 50], showed a lower BCR activity compared to their human RT counterpart, suggests that other BCR-unrelated mechanisms, such as *MYC* overexpression, might modulate BCR activity [51].

RT and RT-PDX cells displayed a high OXPHOS status reveling that mitochondrial metabolism might play a crucial role in response to therapy, considering also that several studies have shown that OXPHOS function is associated to drug resistance [29, 45]. For instance, OXPHOS^high^ activity has been detected in ibrutinib-resistant mantle cell lymphoma patients [52, 53] and recognizes a subtype of DLBCL insensitive to BCR inhibition [54]. OXPHOS inhibitors are promising drugs to misbalance cell metabolism [55]. IACS-010759 is a highly potent and selective small-molecule complex I inhibitor [28] that impairs proliferation and induces apoptosis in brain tumors, acute myeloid leukemia (AML) [29, 30] and *NOTCH1*-mutated T-cell acute lymphoblastic leukemia [56] although the first clinical trial has shown limited antitumor activity [57]. Our results showed that IACS-010759 inhibited proliferation on RT-PDXs characterized by an OXPHOS^high^ phenotype, both in vitro and in vivo, as previously reported [11]. In contrast, CLL cells use different metabolic pathways when OXPHOS is inhibited, being necessary to target OXPHOS and glycolysis to induce apoptosis [58], suggesting a different balance between OXPHOS and glycolysis-based metabolism. Furthermore, OXPHOS inhibition overcame venetoclax resistance in our RT-PDXs models, as it has been reported in AML [29, 30, 59] and in high grade MYC-DLBCL [54]. Furthermore, since it has been reported that CLL cells may impose metabolic alterations on CD4^+^ T cells [60], we can hypothesize that OXPHOS inhibition might lead to the improvement of T-cell based therapies.

Overall, although the generation of PDX models is challenging and with a low success rate, when established, they may represent a tool to understand the clonal evolution of a tumor. Our results showed, for the first time, the generation of a RT-PDX from a CLL sample, demonstrating that RT cells are already present early-in-time within the circulating CLL cells and allowing to study disease transformation. Here we uncovered a remarkable subclonal heterogeneity in RT and confirmed that multiple RT subclones might be present in early stages of the disease. The OXPHOS^high^–BCR^low^ axis observed could contribute to their low response to BCR inhibitors, as observed in RT patients. In this study we propose that targeting OXPHOS in combination with BH3-mimetic inhibitors might be a potentially new therapeutic opportunity for RT patients, characterized by dismal prognosis and limited therapies.

### Supplementary information

Supplemental material

Supplemental tables

## Data Availability

WGS and RNA-seq data have been deposited at the European Genome-phenome Archive (accession number EGAS00001006965). The datasets generated and/or analyzed during the current study are available from the corresponding author on reasonable request.
