# Supplementary material for: Chronic lymphocytic leukemia patient-derived xenografts recapitulate clonal evolution to Richter transformation
Source: Leukemia. 2023 Nov 28;38(3):557–69. doi: 10.1038/s41375-023-02095-5 (PMC10912031; doi:10.1038/s41375-023-02095-5)
Supplement: Supplementary file 1 — Supplemental material [file 41375_2023_2095_MOESM1_ESM.pdf]

Supplemental Materials

Chronic lymphocytic leukemia patient-derived xenografts recapitulate clonal evolution to Richter transformation

Playa-Albinyana H, *et al.*

Supplemental Methods 3

Human cell lines 3
Immunocompromised mice 3
PDX generation 3
Clonality studies 4
In vivo model 4
Whole-genome sequencing (WGS) 5
RNA-seq 7
Fluorescence in situ hybridization (FISH) 8
In vitro studies 8
Immunohistochemistry 9
Western blot analysis 9
Calcium flux analysis 10
Oxygen consumption determination 10
PDX *in vitro* treatments 11
Proliferation assays 12
Cytotoxicity assays 12

Supplemental Figures 13

Figure S1 13
Figure S2 14
Figure S3 15
Figure S4 16
Figure S5 17
Figure S6 18
Figure S7 19
Figure S8 20
Figure S9 21
Figure S10 21
Figure S11 23
Figure S12 22
Figure S13 24

Supplemental Tables 25

Supplemental References 26

Supplemental Methods

Human cell lines

MEC-1 cell line (ACC 497) from the German Collection of Microorganisms and Cell Cultures (DSMZ) and the human bone-marrow derived stromal cell line HS-5 (CRL-11882) from the American Type Culture Collection (ATCC) were used. Mycoplasma contamination in cell lines were routinely tested by PCR. Identification of cell lines was done using the GenePrint 10 system (Promega, Madison, WI, USA). MEC-1 cells (5×10^5 cells/mL) were cultured under Iscove's modified Dulbecco's medium [IMDM] with GlutaMAX™ (Gibco, Waltham, MA, USA) supplemented with 10% (v/v) heat-inactivated fetal bovine serum (FBS) and 1% (v/v) penicillin-streptomycin 10,000 U/mL (Gibco) and HS-5 cells (4×10^4 cells/mL) were cultured in Dulbecco's modified Eagle's medium [DMEM] (Life Technologies). Cells were seeded overnight, and once obtained a confluent stroma monolayer; medium was replaced by CLL cells.

Immunocompromised mice

Four- to eight-month female immunocompromised mice NOD.Cg-*Prkdc*^{scid}*Il2rg*^{tmlWjl}/SzJ (NOD-SCID IL2rγ^{null}; NSG) (Charles Rivers Laboratories) were housed under restricted pathogen-free conditions in accordance with the *Principles of Laboratory Animal Care* and approved by the University of Barcelona (Departament de Territori i Sosteniblitat, Generalitat de Catalunya, authorization #9680). Mice were used for tumor engraftment.

PDX generation

T cells from cryopreserved PBMCs of CLL or RT samples were isolated and purified using a positive selection through human CD2 microbeads (Miltenyi Biotec, Bergisch Gladbach, Germany). Then, these T cells were *in vitro* activated for 24h with anti-CD3/CD28 Dynabeads® (ThermoFisher Scientific) and 30 IU/mL human IL-2 (R&D Systems, Minneapolis, MN, USA). In the meantime, thawed CLL cells were maintained with 0.2µM ODN2006-TL9 (InvivoGen) and 15 ng/mL human IL-15 (R&D Systems). When tumor masses were palpable (≥1.5 cm^3), mice were sacrificed, tumors disrupted and $5x10^5$ of cells reinjected SC into a new mouse. This process was repeated until PDXs were established.

Clonality studies

Tumor cells were isolated and human cells were purified using human CD19 (hCD19) microbeads (Miltenyi Biotec). DNA from purified PDXs was extracted using QIAamp® DNA Mini Kit (Qiagen, Hilden, Germany) according to manufacturer's protocol and quantified using the Qubit spectrophotometer (ThermoFisher Scientific). DNA was amplified using primers corresponding to the variable heavy framework FR1 region of IGH using the BIOMED-2 multiplex PCR protocol[1] and analyzed by fragment analysis using Genemapper Software. Images were compared with the original clonal rearrangement of each case.

***In vivo* model**

<u>*In vivo model generation*</u>. 10^7 cells from PDXs were resuspended in media and injected IV in the tail of NSG mice. Engraftment was monitored by optical *in vivo* imaging system IVIS® (PerkinElmer, Waltham, MA, USA) using fluorescent probe XenoLight RediJect 2-DG-750 (PerkinElmer). Cell infiltration was calculated as radiant efficiency ($\frac{p/sec/cm^2/sr}{\mu W/cm^2}$) with a scale from hot to cold colors as higher to lower infiltration. Peripheral blood (PB), spleen and bone marrow (BM) were collected and tumoral cells ($CD19^+$ $CD5^+$) were evaluated by flow cytometry: hCD45 (Pacific Blue); Annexin V (FITC); hCD19 (SuperBright600) [Invitrogen]; hCD3 (PerCP/Cy5.5); mCD45 (PE-Cy7) and hCD5 (PE-Cy5) [BD Biosciences] (Table S2). A minimum of 0.5 x 10^6 cells were resuspended in Annexin V-Binding Buffer (10mM HEPES [Sigma-Aldrich, Saint Louis, MO, USA]; 140mM NaCl [EMSURE] and 2.5 mM $CaCl_2$ [Sigma-Aldrich] in deionized water and adjusted a pH 7.4) and labelled with the respective surface marker antibody cocktails for 30 minutes at 4ºC followed by a wash with 1 mL of Annexin V-Binding Buffer. Cells were acquired in a LRSFortessa™ (BD Biosciences) using the BD FACSDiva software v8. Analysis of cell population was conducted using FlowJo v10 (TreeStar, Ashland, OR, USA). Gating strategy was as follows: cell identification in FSC-A vs. SSC-A plot; singlets identification in FSC-A vs. FSC-H plot; human leukocytes cells in hCD45 (Pacific Blue) vs. mCD45 (Pe-Cy7) plot; tumoral cells were identified from $hCD45^+$ cells in hCD19 (SuperBright600) vs. hCD5 (PE-Cy5) plot; finally, T cells were identified from $hCD45^+$ cells in hCD3 (PerCP/Cy5.5) vs. SSC-A plot.

<u>*In vivo drug studies*</u>. To test the efficacy of IACS-010759, 10 NSG mice were randomized by age and weight prior PDX injection. The day after, 10^7 PDX12-P8 cells

were injected IV and let them spread. After 7 days, 4 mice (Control) received 0.5% methylcellulose (once daily) as vehicle and 6 mice received IACS-010759 (Selleck Chemicals LLC, Houston, TX, USA) (5 mg/kg, once daily) via oral gavage. Mice were weighted every 2 days and engraftment was monitored by optical *in vivo* imaging system IVIS® (PerkinElmer) using fluorescent probe XenoLight RediJect 2-DG-750 (PerkinElmer). To confirm the efficacy of combining IACS-010759 with venetoclax, 16 NSG mice were randomized by age and weight prior PDX injection. The day after, 107 PDX12-P8 cells were injected IV and let them spread. After 7 days, 4 mice (Control) received 0.5% methylcellulose (once daily) as vehicle, 4 mice (venetoclax) received 25mg/kg of venetoclax (Selleck Chemicals LLC) (once daily), 4 mice (IACS) received 5mg/kg of IACS-010759 (Selleck Chemicals LLC) (once daily) and finally, 4 mice (Venetocax + IACS) received 25mg/kg of venetoclax combined with 5mg/kg of IACS-010759 (once daily), all of them via oral gavage. Mice were weighted every 2 days and engraftment was monitored by optical *in vivo* imaging system IVIS® (PerkinElmer) using fluorescent probe IRDye ® 800CW 2-DG Optical Probe (LI-COR Biosciences, Lincoln, NE, USA).

Whole-genome sequencing (WGS)

Library preparation and sequencing. Purified DNA from PDX samples were subjected to WGS (Macrogen, Seoul, South Korea). Libraries were performed using the TruSeq DNA PCR-Free kit (Illumina, cat. no. 20015963) starting with 1µg of input DNA and performed following manufacturer's instructions. Libraries were sequenced on a NovaSeq 6000 (2x151 bp) instrument (Illumina). A mean coverage of 30.4x was obtained. We also included previously published WGS data and results (case 12: germline sample, original CLL, and RT evolving in the patient; case 19: germline and original RT sample) (Table S3).[2]

Read mapping, filtering of reads of potential mouse origin and quality control. Raw reads were aligned using the BWA-MEM algorithm (v0.7.15).[3] BAM files were generated, and optical/PCR duplicates flagged using biobambam2 (v2.0.65, https://gitlab.com/german.tischler/biobambam2). For PDX samples, raw reads were mapped in parallel to both the human (GRCh37) and *mus musculus* (GRCm38) reference genomes. Disambiguate (v1.0.0),[4] which separates sequencing reads of two species derived from grafted samples, was used to distinguish reads of human and mouse origin. Only human reads were used for downstream analyses. FastQC

(v0.11.5, www.bioinformatics.babraham.ac.uk/projects/fastqc) and Picard (v2.10.2, https://broadinstitute.github.io/picard) were used to extract quality control metrics.

IG gene characterization. IG gene rearrangements were characterized using IgCaller (v1.2)[5] and the obtained sequences were reviewed on the Integrative Genomics Viewer (IGV, v2.9.2),[6] and annotated using IMGT/V-QUEST and ARResT/AssignSubsets online tools (Table S4).

Variant calling. Variant calling was performed as previously described.[2] Briefly, single nucleotide variants (SNV) were called using CaVEMan (cgpCaVEManWrapper, v1.12.0),[7] Mutect2 (GATK v4.0.2.0)[8] and MuSE (v1.0 rc),[9] and normalized using bcftools (v1.8).[10] Variants detected by CaVEMan with CLPM>0 and ASMD values <140 were excluded. Variants called by Mutect2 with MMQ<60 were eliminated. Only mutations detected by at least two algorithms were considered. Short insertions/deletions (indels) were called by SMuFin (v0.9.4),[11] Pindel (cgpPindel, v2.2.3),[12] SvABA (v7.0.2),[13] Mutect2 (GATK v4.0.2.0),[8] and Platypus (v0.8.1).[14] The somaticMutationDetector.py script(https://github.com/andyrimmer/Platypus/blob/master/extensions/Cancer/somaticMutationDetector.py) was used to identify somatic indels called by Platypus. Indels were left-aligned and normalized using bcftools.[10] Indels with MMQ<60, MQ<60, and MAPQ<60 for Mutect2, Platypus, and SvABA, respectively, were removed. Only indels identified by at least two algorithms were retained. snpEff/snpSift (v4.3t)[15] was used to annotate the mutations identified using GRCh37.p13.RefSeq as a reference. Copy number alterations (CNA) were called using Battenberg (cgpBattenberg, v3.2.2)[16] and ASCAT (ascatNgs, v4.1.0).[17] CNA within any of the immunoglobulin loci were not considered. Structural variances (SVs) were extracted using SMuFin (v0.9.4),9 BRASS (v6.0.5),[18] SvABA (v7.0.2),[13] and DELLY2 (v0.8.1).[19] SVs identified were intersected considering a window of 300 bp around breakpoints and only those identified by at least two programs, when at least one of the algorithms called the alteration with high quality (MAPQ≥90 for BRASS, MAPQ=60 for SvABA and DELLY2), were used in downstream analyses. IgCaller (v1.2)[5] was also used to call SVs within any of the immunoglobulin loci. All SVs were visually inspected using the Integrative Genomics Viewer (IGV).[6]

Subclonal reconstruction. SNV called in one sample were automatically added to the samples of additional time point(s) if at least one high-quality read with the mutation was found in the BAM file (alleleCounter v4.0.0, parameters: min_map_qual=35, min_base_qual=20), as previously described.[2] Similarly, indels

detected in one sample were added in the additional time point(s) if any of the algorithms detected the alteration, regardless of its filters.[2] Next, a Markov chain Monte Carlo sampler for a Dirichlet process mixture model was used to infer putative subclones, to assign mutations to subclones and to estimate the subclone frequencies in each sample from the SNV read counts, copy number states and tumor purities.[2, 16, 20] The phylogenetic relationships between subclones were identified following the "pigeonhole principle", which was relaxed using a case-specific "tolerated error" (Table S9).[2, 20] Clusters not assigned to the reconstructed phylogenetic tree were excluded. The subclonal reconstruction obtained here using the set of samples included in this study was adjusted considering that of our previous study,[2] which included additional CLL samples and allowed us to better discriminate the mutations assigned to two subclones, one of each case (Table S10). CNA and indels affecting potential driver genes were manually assigned to the most likely subclone based on their cancer cell fraction (CCF) in the different timepoints analyzed. Fish plots were generated using the TimeScape R package (v1.6.0).

Mutational signatures. Mutational signatures were analyzed for SNV or single base substitutions (SBS) according to their 5' and 3' flanking bases. We measured the contribution of mutational signatures previously identified in CLL and RT (SBS1, SBS5, SBS8, SBS9, SBS18, and SBS-RT)[2] in the mutational profile of each reconstructed subclone using a fitting approach (MutationalPatterns, v3.4.1). To avoid the so-called inter-sample bleeding effect,[21] we iteratively removed the less contributing signature if its removal decreased the cosine similarity between the original and reconstructed 96-profile <0.01.[21] SBS1 and SBS5 were added if addition improved the cosine similarity.[22]

RNA-seq

Data generation. Bulk RNA-seq data of 8 RNA samples were generated. Libraries were prepared using the Stranded mRNA Library Prep, Ligation Kit (Illumina, cat. no. 20040534) following manufacturer's recommendations and sequenced on a NextSeq 2000 (2x50 bp, Illumina). The raw data of 3 samples (case 12: CLL sample prior to therapy and RT sample evolving in the patient; case 19: CLL sample prior to therapy) were obtained from a previous publication[2] and reanalyzed for harmonization purposes (Table S12).

Data analysis. Ribosomal RNA reads were filter out using SortMeRNA (v4.3.4, database used: smr_v4.3_default_db.fasta).[23] Non-ribosomal reads were trimmed using trimmomatic (v0.40).[24] Quality control of the original and trimmed FASTQ files was performed using FastQC (v.0.11.9). Gene-level counts (GRCh38.p13, Ensembl release 105) were calculated using kallisto (v0.46.1)[25] and tximport (v1.22.0). The DESeq2 (v1.34.0)[26] workflow was used to normalize the expression counts. Sample-wise gene set enrichment scores were calculated using Gene Set Variation Analysis (GSVA) (v1.42.0, method: gsva)[27] considering C2 (curated), C5 (ontology), and H (hallmark) gene sets from the Molecular Signatures Database (MSigDB, v7.5.1, minimum size = 10, maximum size = 250). These analyses were performed in R (v.4.1.3).

Fluorescence in situ hybridization (FISH)

FISH studies were performed on fixed PDX cells. Interphase nuclei were hybridized with commercially available probes: LSI XL MYC (8q24) dual color break-apart (Metasystems, Heidelberg, Germany). Hybridization and detection were performed according to the manufacturer's protocols. Translocations were detected by fluorescence microscopy after nuclear counterstaining with DAPI (4',6 diamidino-2-phenylindole).

***In vitro* studies**

PDX cells were seed (10^6 cells/mL) in a flat or round bottom 96-well cell culture plate on enriched medium (EM) containing RPMI-1640 (Gibco) supplemented with 1% Glutamax (Gibco), 15% FBS (Gibco), 5.5X Insulin-Transferrin-Selenium (ITS) (Sigma-Aldrich), 1X non-essential amino acids (Gibco), 1X sodium pyruvate (Gibco), 10mM HEPES (Fisher), 50µg/mL gentamycin-sulfate (Gibco) and 1X β-mercaptoethanol (Gibco) as previously described.[28, 29] or on RPMI-1640 with 1% Glutamax, 10% FBS and 5% penicillin [10,000 IU/mL] / streptomycin [10 mg/mL] (ThermoFisher Scientific) with or without HS-5 bone marrow stromal cells in a proportion of 4:1 or with or without 0.2µM ODN2006-TL9 (InvivoGen) and 15ng/mL human IL-15 (R&D Systems)[28]. Cells were incubated at 37ºC and 5% CO_2 or on RPMI-1640 (Gibco) with 1% Glutamax (Gibco), 10% FBS (Gibco) and 5% penicillin [10,000 IU/mL] / streptomycin [10 mg/mL] (ThermoFisher Scientific) with or without HS-5 bone marrow stromal cells in a

proportion of 4:1 or with or without 0.2µM ODN2006-TL9 (InvivoGen) and 15ng/mL human IL-15 (R&D Systems).[28] Cells were incubated at 37ºC and 5% CO_2.

Immunohistochemistry

Mice spleens were formalin-fixed paraffin-embedded and characterized with a panel of immunohistochemical antibodies including: CD79a [clone SP18] and CD3 [clone 2GV6; Roche Diagnostics] (human) and F4/80 [clone CI:A3-1; Bio-Rad Laboratories, Hercules, CA, USA] (mouse). Three-micrometer-thick sections were used in a peroxidase-labeled detection system, standard antigen retrieval protocols and an automated immunostainer (AutostainerLink 48 [Dako, Carpinteria, CA, USA], or Benchmark Ultra [Roche Diagnostics, Basel, Switzerland]) were performed as previously described.[30]

Western blot analysis

Whole-cell protein extraction was obtained from hCD19 PDXs purified cells with RIPA buffer (Sigma-Aldrich) supplemented with cOmpletecomplete™ ULTRA Tablets, Mini, EASYpack Protease Inhibitor Cocktail and PhosSTOP™ (Roche). Proteins were quantified by Lowry modified (ThermoFisher Scientific) according to manufacturer's protocol, separated in 4–20% Mini-PROTEAN® TGX Stain-Free™ (Bio-Rad) polyacrylamide gel and transferred to an Immobilon-P PVDF membrane (EMD Millipore, Burlington, MA, USA). Membranes were blocked with 2.5% phosphoBlocker Blocking Reagent (Cell Biolabs, San Diego, CA, USA) in Tris-Buffered Saline (TBS) - 0.1% Tween 20. We probed with antibodies against c-Myc (D84C12), TAK1 (D94D7) (Cell Signaling, Danvers, MA, USA) and Bcl-2 (100) (Santa Cruz Biotechnology, Dallas, TX, USA). Antibody binding was detected using secondary peroxidase-labeled anti-mouse and anti-rabbit antibodies (Cell Signaling) and chemiluminescence was detected using a mini-LAS4000 Fujifilm device (Fujifilm, Tokyo, Japan). Equal protein loading was confirmed by probing membranes with β-actin antibody (Sigma).

Calcium flux analysis

Measurement of Ca^{2+} flux was performed as previously described.[31] PDX cryopreserved cells were thawed, counted, and resuspended on RPMI-1640 (Gibco) with 1% Glutamax (Gibco), 10% FBS (Gibco) and 5% penicillin [10,000 IU/mL] / streptomycin [10 mg/mL] (ThermoFisher Scientific) at 10^6 cells/mL and incubated during six hours at 37ºC and 5% CO_2. After, cells were centrifuged and resuspended on RPMI-1640 (Gibco) with 4 µM Indo-1 AM (ThermoFisher Scientific) and 0.08% Pluronic F-127 (ThermoFisher Scientific) for 30 minutes at 37ºC and 5% CO_2. After, were labelled for 20 minutes, at room temperature, surface marker antibodies: hCD19 (SuperBright600) (Invitrogen) and hCD5 (PE-Cy5) (BD Biosicences) for tumoral cell identification ($CD19^+$ $CD5^+$). Finally, cells were resuspended on RPMI-1640 (Gibco) prior flow cytometry acquisition. Basal calcium was measured during one minute before stimulation. Then, cells were incubated during two minutes at 37ºC with or without 10 µg/mL anti-human F(ab')2 IgM (Southern Biotech, Birmingham, AL, USA) and 3.3 mM H_2O_2 (Sigma-Aldrich). Finally, 2 µM 4-hydroxytamoxifen (4-OHT) (Sigma-Aldrich) were added to cells in all conditions, before continue recording up to 500 seconds. Intracellular Ca^{2+} release was measured in a LRSFortessa™ (BD Biosciences) using the BD FACSDiva software v8 by exciting with ultraviolet laser (355 nm) and appropriate mirrors: Indo-1 violet (450/50 nm) and Indo-1 blue (530/30 nm). To determine the calcium flux, ratio of bound (Indo-1 violet) and unbound (Indo-1 blue) (ratiometrics) was calculated with FlowJo v10 software using kinetics tool. Gating analyses were as follows: cell identification in FSC-A vs. SSC-A plot; singlets identification in FSC-A vs. FSC-H plot, tumoral cells ($CD19^+$ $CD5^+$) in CD19 (SuperBright600) vs. CD5 (PE-Cy5) plot and Ca^{2+} release in Time vs. Indo-1 (violet) / Indo-1 (blue) plot using kinetics tool (Table S13).

Oxygen consumption determination

PDX cryopreserved cells were thawed, counted, and resuspended on RPMI-1640 (Gibco) with 10% FBS (Gibco) and 1% Glutamax (Gibco) (full medium) at a concentration of $3x10^6$ cells/mL. After one hour of incubation at 37ºC and 5% CO_2, cellular respiration in intact cells was performed using O2k-respirometers (Oroboros Instruments GmbH, Innsbruck, Austria). 2 mL of cell suspension were added in each respirometer chamber, at 37ºC at a stirrer speed of 750 rpm. Routine respirations were done by analyzing oxygen consumption in living cells resuspended on full medium. Oligomycin (2µL/mL) (Sigma-Aldrich) was added to inhibit ATP synthase and assess

leak respiration. To measure uncoupler-stimulated electron transfer capacity (ETC) carbonyl cyanide-p-trifluoromethoxyphenylhydrazone (FCCP) (0.5µM) (Sigma-Aldrich) was added sequentially until maximum capacity was reached. To determine residual oxygen consumption, ETS was inhibited by adding sequentially rotenone (R) (0.5µM) (Sigma-Aldrich) and antimycin A (AA) (2.5µM) (Sigma-Aldrich). Data acquisition and real-time analyses were performed using the software DatLab 7.4 (Oroboros Instruments GmbH). Automatic instrumental background corrections were applied for oxygen consumption by the polarographic oxygen sensor and oxygen diffusion into the chamber. The same experimental workflow was used to study cellular respiration after one hour of treatment with IACS-010759 at 100 nM (Tables S14, S15).

PDX *in vitro* treatments

PDX cells were seeded (10^6 cells/mL) in a flat bottom 96-well cell culture plate on enriched medium as previously described[28, 29] or on RPMI-1640 (Gibco) with 1% Glutamax (Gibco), 10% FBS (Gibco) and 5% penicillin [10,000 IU/mL] / streptomycin [10 mg/mL] (ThermoFisher Scientific) with or without HS-5 bone marrow stromal cells in a proportion of 4:1 and with or without 0.2 µM ODN2006-TL9 (InvivoGen) and 15 ng/mL human IL-15 (R&D Systems). Cells were incubated at 37ºC and 5% CO_2. PDX cells were seeded in a round bottom 96-well cell culture plate on enriched medium with 0.2 µM ODN2006-TL9 (InvivoGen) and 15 ng/mL human IL-15 (R&D Systems) and exposed to drugs for the indicated time-point depending on the considered read-out: venetoclax (ABT-199; Selleck Chemicals LLC), 48 hours: 25, 50 or 75nM; ibrutinib (PCI-32765; Selleck Chemicals LLC), three or six days: 1 or 5 µM and IACS-010759 (Selleck Chemicals LLC), 48 or 72 hours: 50, 100 or 150nM; rapamycin (Selleck Chemicals LLC), 48 hours: 1µM; everolimus (RAD-001; Novartis, Basel, Switzerland), 48 hours: 5µM and 2-deoxyglucose (2-DG; Sigma-Aldrich), 48 hours: 5mM. After drug treatment, cells were processed by flow cytometry (Fig. S13A).

Proliferation assays

PDX cells were labeled with 0.5 µM carboxyfluoresceinsuccinimidyl ester (CFSE Cell Tracer; ThermoFisher Scientific). After labeling, cells were seeded at a density of 10^6 cells/mL and treated with ibrutinib and/or IACS-010759. The percentage of proliferating cells was determined six days after ibrutinib or 72 hours after IACS-010759 treatment on LRSFortessa™ (BD Biosciences) using the BD FACSDiva software v8. Results were analyzed with FlowJo v10 software. Gating analyses were as follows: cell identification in FSC-A vs. SSC-A plot; singlets identification in FSC-A vs. FSC-H plot, alive cells in Annexin-V (Pacific Blue) vs. SSC-A plot, tumoral cells ($CD19^+$ $CD5^+$) in CD19 (SuperBright600) vs. CD5 (PE-Cy5) plot and cell proliferation in CFSE histogram. Proliferation was determined as percentage of CFSE low cell expression as reported previously (Fig. S13B, C).[32]

Cytotoxicity assays

Cells incubated with venetoclax and/or IACS-010759 were stained with Annexin-V (Pacific Blue) (Invitrogen), CD19 (SuperBright600) (Invitrogen) and CD5 (PE-Cy5) (BD Bioscience), see Table S2 for antibodies details. Labeled samples were acquired on LRSFortessa™ (BD Biosciences) using the BD FACSDiva software v8. Tumor cells were identified by gating double positive population. Percentage of viable cells was determined as $Annexin\text{-}V^-$ (Pacific Blue) cells after 48 or 72 hours. Results were analyzed with FlowJo v10 software. Gating analyses were as follows: cell identification in FSC-A vs. SSC-A plot; singlet identification in FSC-A vs. FSC-H plot, tumoral cells ($CD19^+$ $CD5^+$) in CD19 (SuperBright600) vs. CD5 (PE-Cy5) plot and alive cells in Annexin-V (Pacific Blue) vs. SSC-A plot.

Supplemental Figures

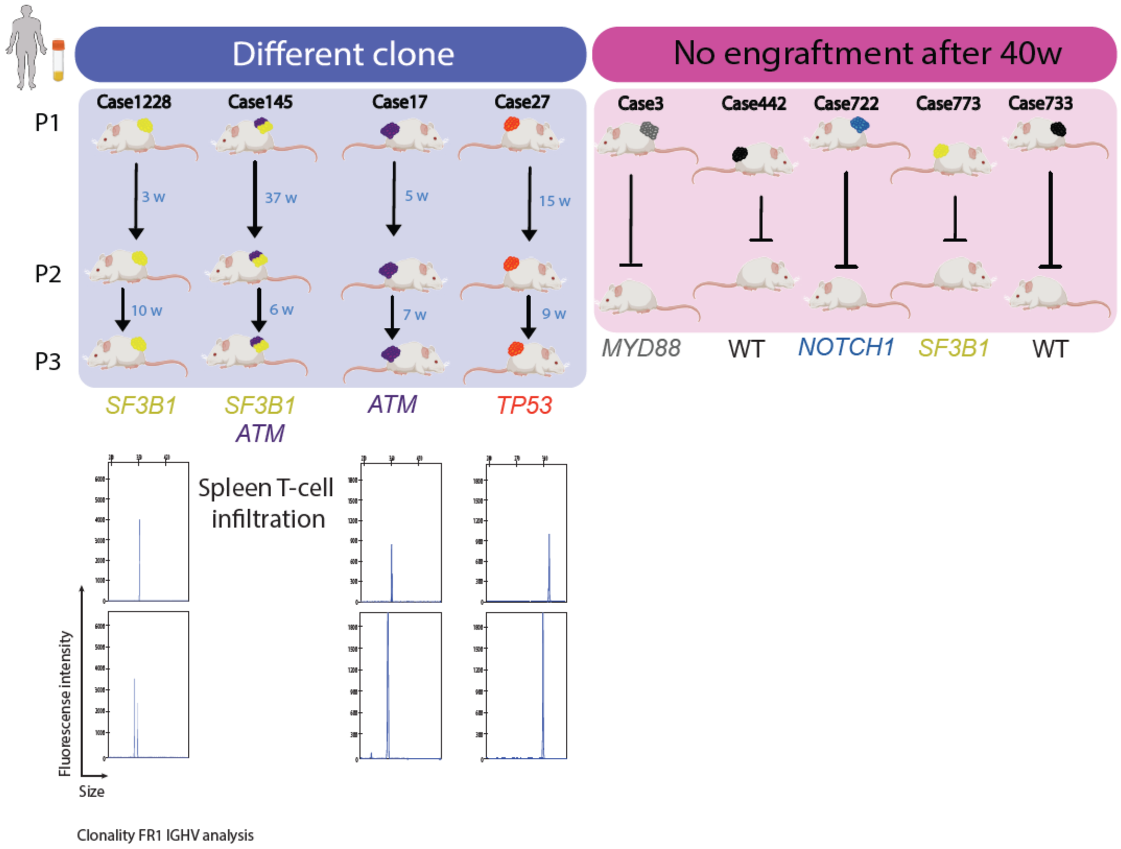

Fig. S1. Schematic representation of all PDX generation attempts. Primary CLL or RT samples from PB were SC injected in NSG mice and let them to engraft until tumor masses were palpable (≥1.5 cm^3). Four cases engrafted a nonrelated CLL B-cell clone (blue box). Whereas five cases (pink box) did not engraft after 40 weeks. Main mutations are indicated in mice with their color code. Time between passages is indicated in blue. For cases with a different clone, clonality results are shown under each case.

A

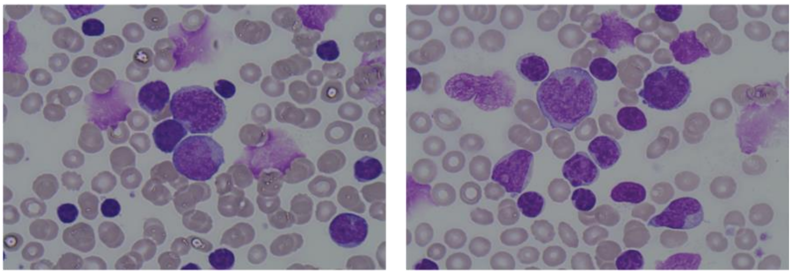

B

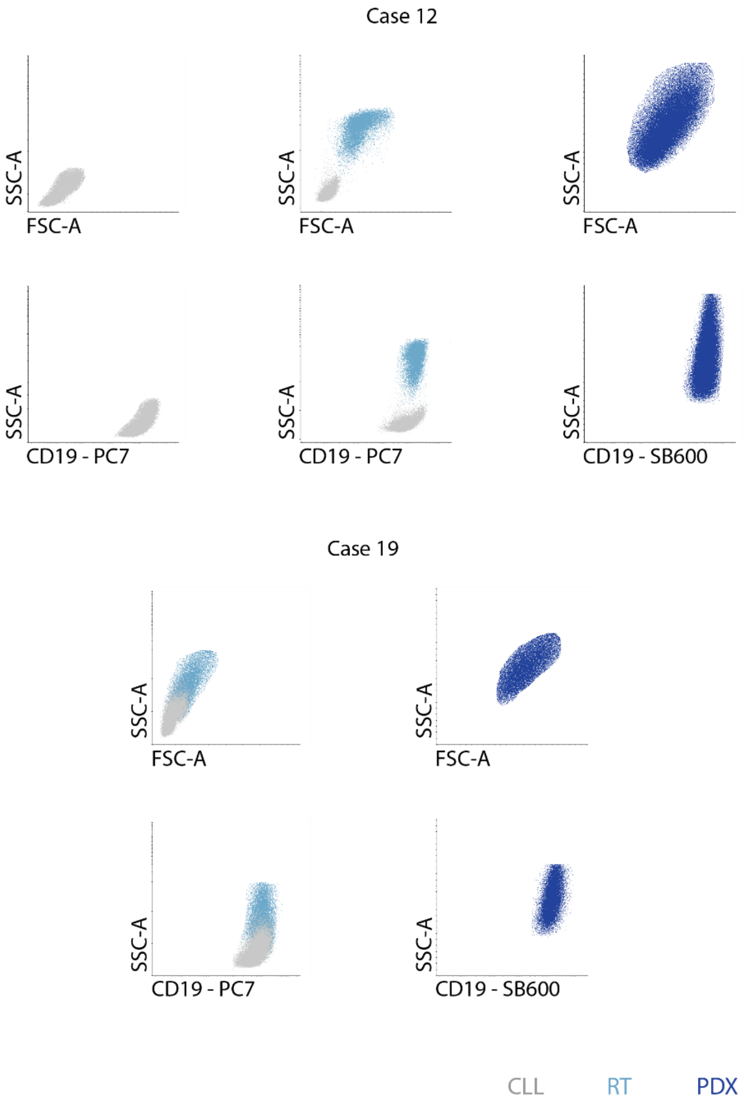

Fig. S2. Characterization of CLL and RT cells. (**A**) Morphology of peripheral blood from RT cases stained with May-Grünwald Giemsa. Around 20-50% of large, very atypical B-cells with centroblastic and immunoblastic morphology were observed. Images were acquired at ×100 magnification. **(B)** Characterization of CLL, RT and PDX samples from case 12 and case 19 by flow cytometry. Size (FSC-A) and complexity (SSC-A) where compared among them as well as the expression of CD19 labeled with PC7 or SB600.

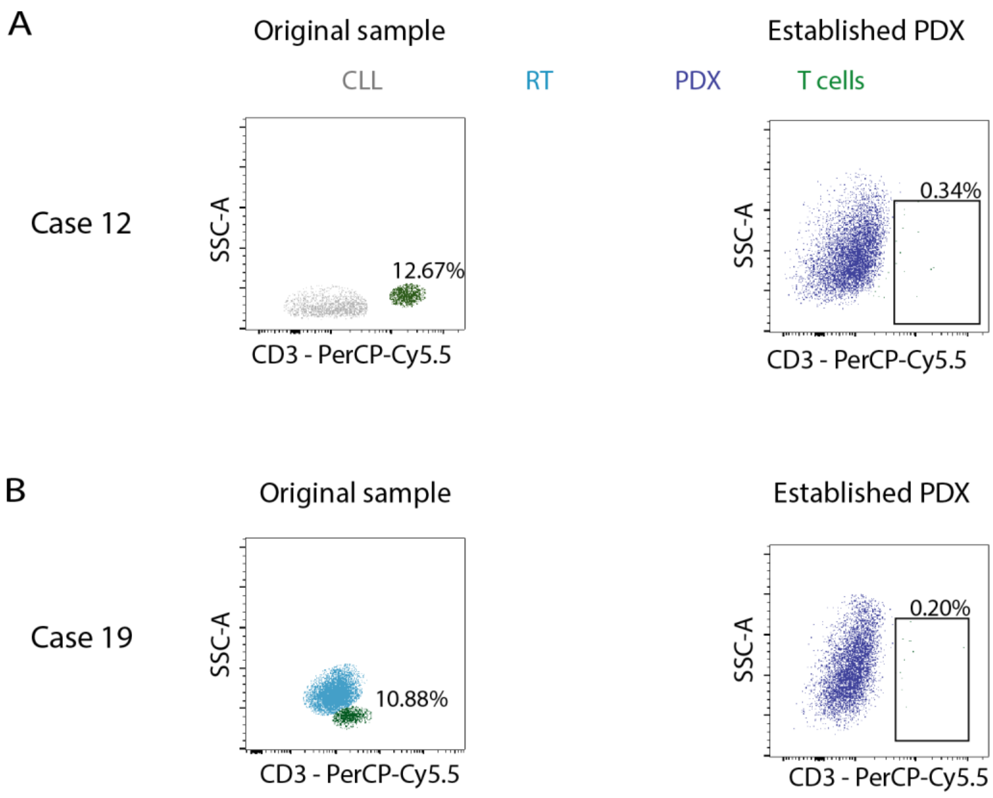

Fig. S3. Loss of T-cell population in PDX samples. A T-cell expression in the original (CLL12) sample (green) together with CLL cells (gray). After PDX (dark blue) is established, T cells disappears as shown in the graph with less than 1% of expression. **B** T-cell expression in the original (RT19) sample (green) together with RT cells (light blue). After PDX (dark blue) is established, scarce T cells were detected as shown in the graph with less than 1% of cells expressing CD3.

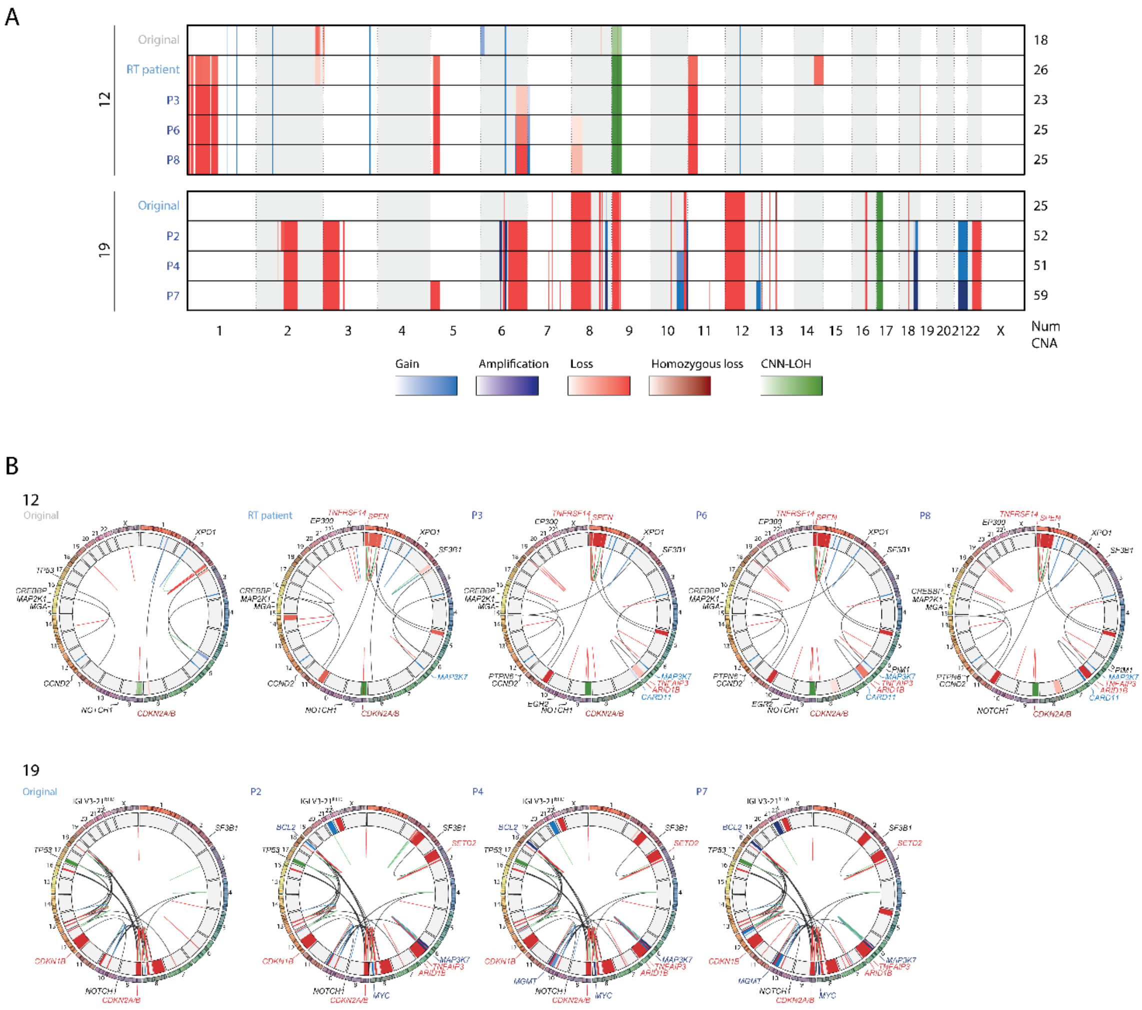

Fig. S4. Genomic landscape of PDX samples. A CNAs found in each PDX (dark blue) and original CLL (gray) and/or RT (light blue) samples according to WGS analyses. Samples are grouped by case. **B** Circos plots illustrating the CNAs and SVs of both cases. Chromosomes are displayed in the outer circle. The following section shows the CNAs painted according to their type. The transparency of the color is proportional to their CCF. The links represents the SVs, linking together the breakpoints of the affected loci. Candidate driver genes affected by CNAs, SVs and/or mutations are annotated.

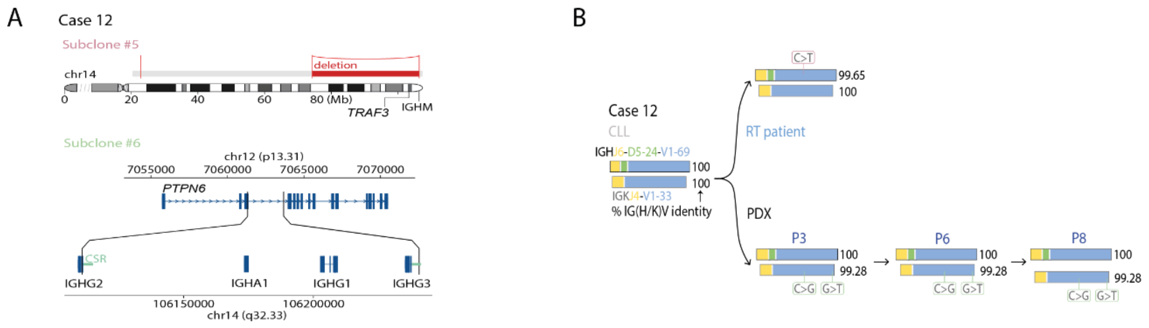

Fig. S5. Convergent evolution in PDX12. A Representation of alterations affecting the immunoglobulin heavy chain (IGH) loci and mediated by aberrant class-switch recombination in subclones #5 and #6 affecting *TRAF3* and *PTPN6*, respectively. **B** Schematic representation of the mutations found in the immunoglobulin genes in the samples analyzed for case 12. RT sample acquired a mutation (C>T) in the IGH not present in CLL and PDX samples. Contrarily, PDX acquired two mutations (C>G and G>T) in the kappa light chain not present in patient's samples.

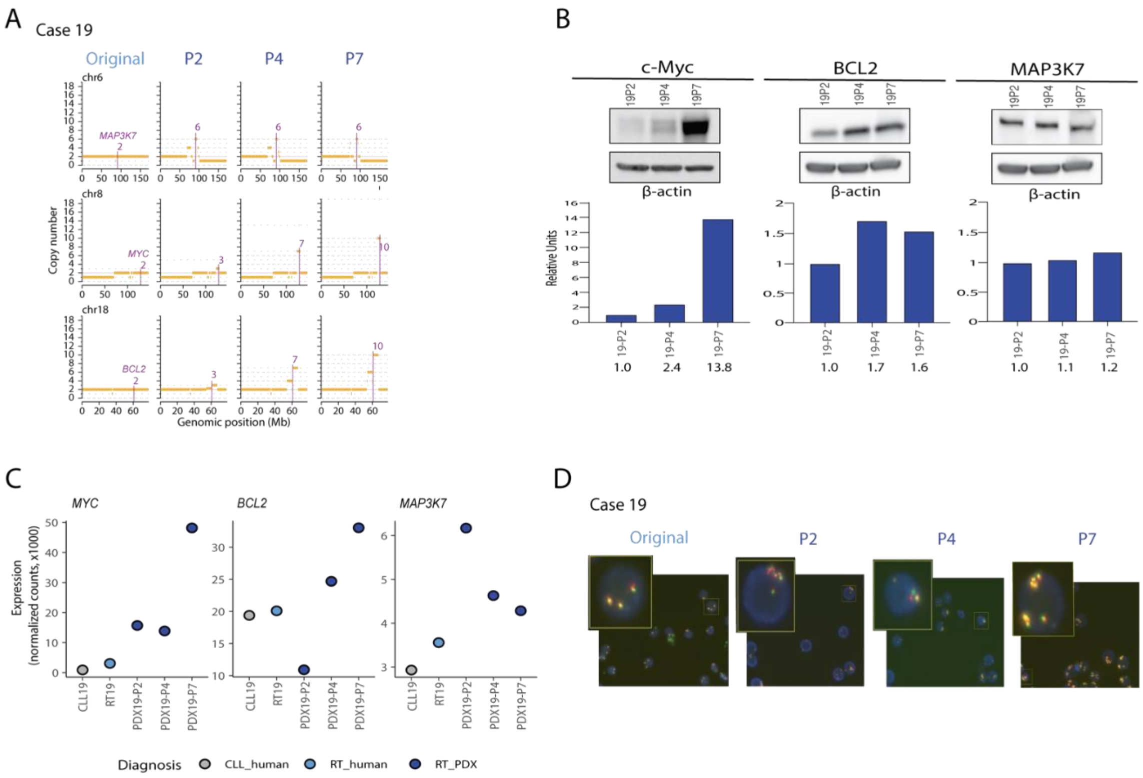

Fig. S6. PDX19 validation of *MYC*, *BCL2* and *MAP3K7* amplifications. A Copy number of *MYC*, *BCL2* and *MAP3K7* detected by WGS. In pink is indicated the number of copies in original RT sample and each PDX passage. **B** Western blot validation of MYC, BCL2 and MAP3K7 in P2, P4 and P7. Quantification was normalized using the housekeeping β-actin gene and quantified (bar chat bottom). **C** RNA-seq expression data. Normalized counts of *MYC*, *BCL2* and *MAP3K7* are depicted for all samples. **D** FISH dual color break-apart probe to detect *MYC* amplification. The amplification is detected in higher percentage of cells over the passages.

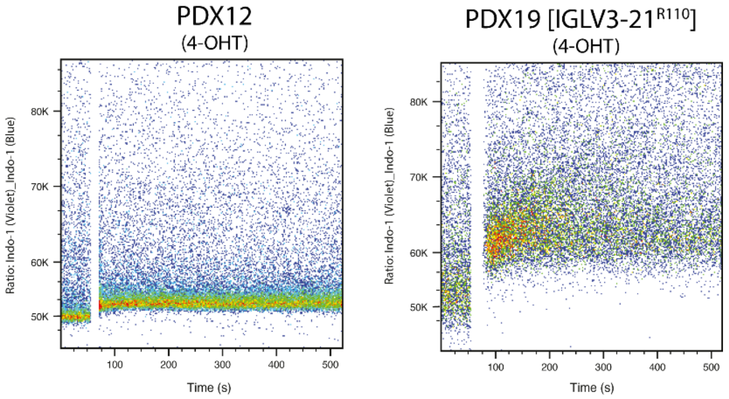

Fig. S7. IGLV3-21^{R110} detection by flow cytometry. Ca^{2+} release kinetics by flow cytometry for PDX12 cells lacking the IGLV3-21^{R110} mutation [left] and for PDX19 carrying the IGLV3-21^{R110} mutation [right] upon incubation with 4-OHT without IgM stimulation. Cells from PDX19 showed an autonomous BCR signaling in line with the presence of the IGLV3-21^{R110} mutation, not detected in PDX12.

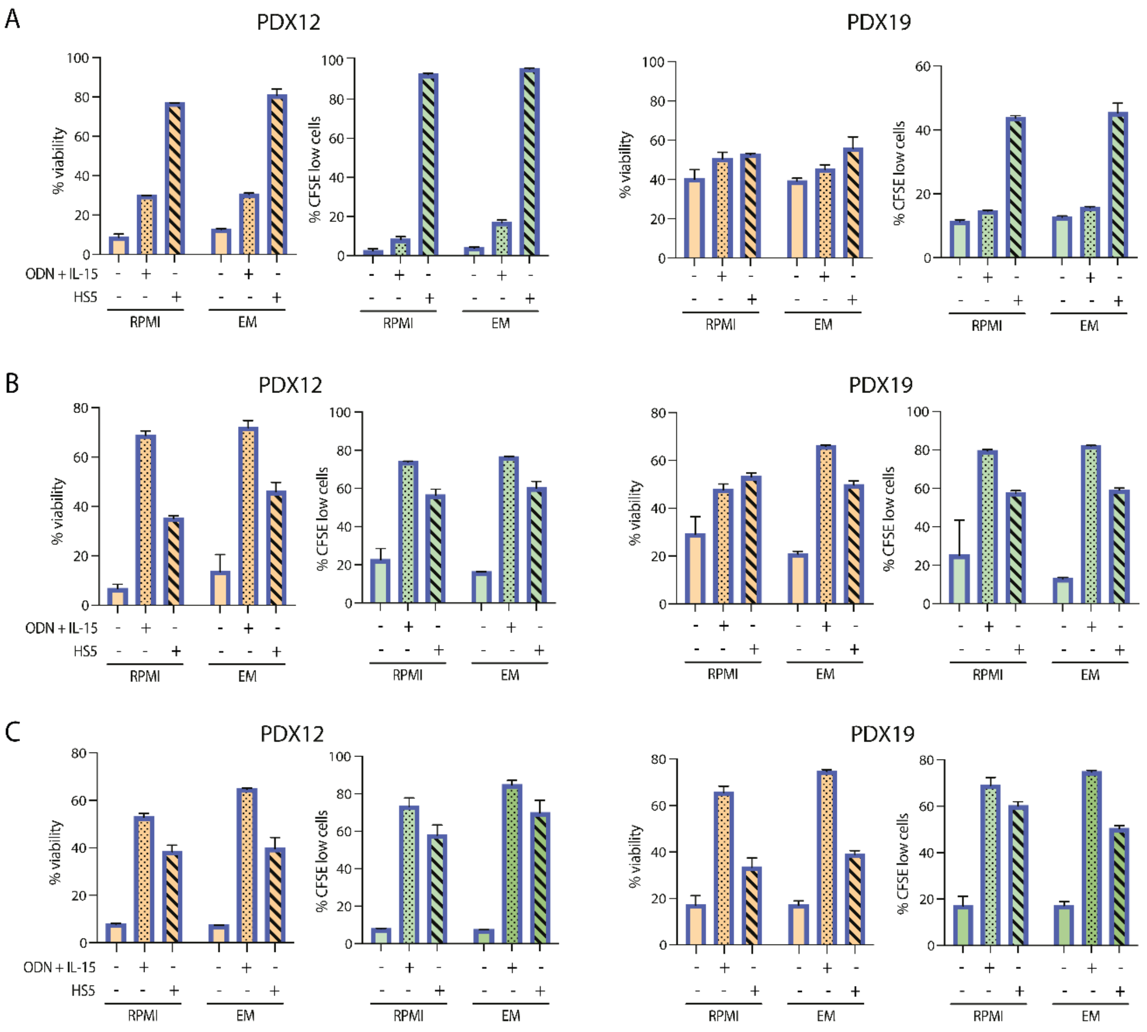

Fig. S8. *In vitro* cell culture conditions. A 24 hours of cell culture from PDXs 12 and 19. Cells were cultured with complete RPMI or enriched medium (EM) medias and without stimuli or soluble stimuli (ODN2006 + IL-15) or stromal cell line (HS-5). Cell viability was determined as percentage of viable cells measured by Annexin-V staining and proliferation was determined using carboxyfluorescein succinimidyl ester (CFSE) cell tracer. Errors bar: SD (n=3). **B** 48 hours of cell culture from PDXs 12 and 19 under same conditions from panel A. **C** 72 hours of cell culture from PDXs 12 and 19 under same conditions from panel A.

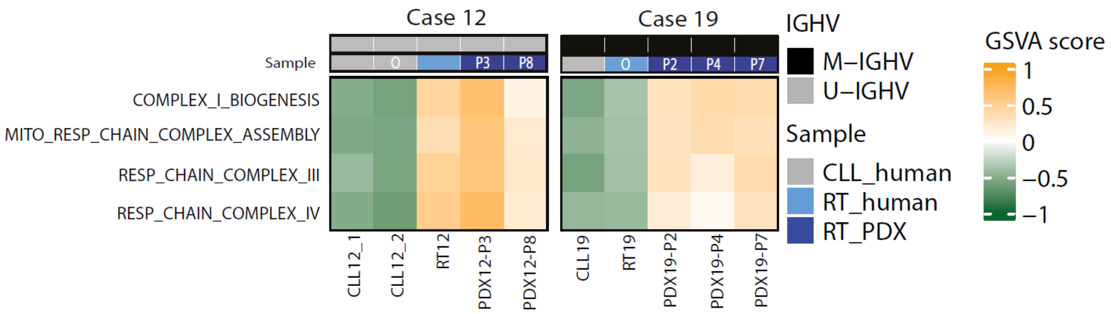

Figure S9. Mitochondrial complexes RNA expression. Heat map showing the gene set enrichment scores (GSVA score) of mitochondrial complexes from original CLL/RT and PDX samples from both cases. Complex I is from Human MSigDB Collections C2: curated gene sets and rest of the complexes are from Human MSigDB Collections C5: ontology gene sets.

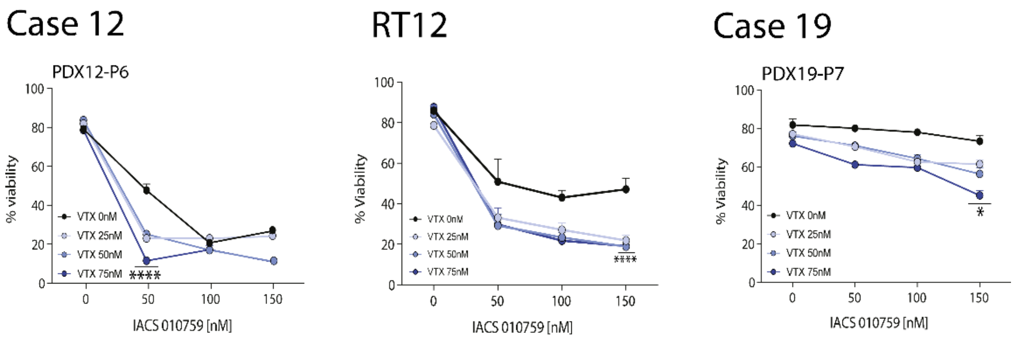

Figure S10. IACS-010759 circumvent venetoclax resistance in PDX cells. Percentage of alive (Annexin V$^-$) PDX12, RT12 and PDX19 cells after 48h of treatment with the corresponding concentrations of IACS-010759 and venetoclax (VTX). Error bars: SD (n = 3).

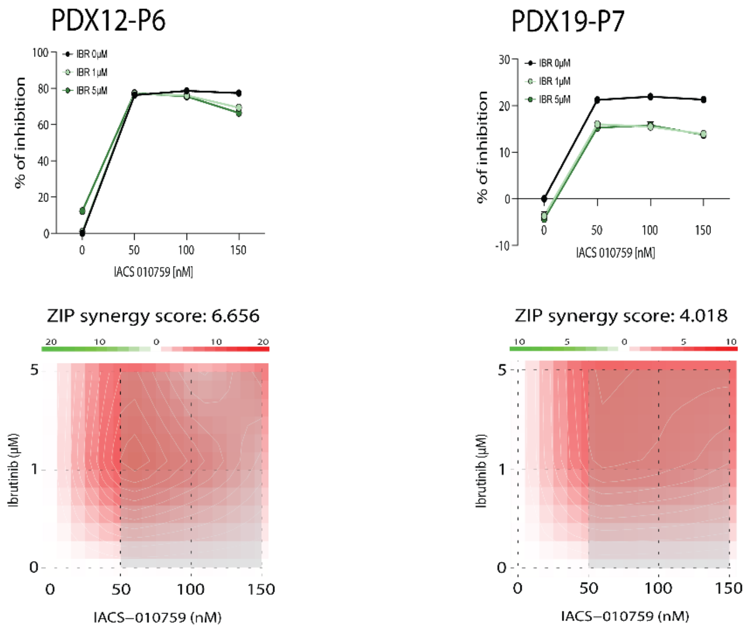

Figure S11. IACS-010759 in combination with ibrutinib. Percentage of growth inhibition after 72 hours of treatment with the indicated concentrations of IACS-010759 and ibrutinib (IBR) in PDX12. Drug interaction landscape and synergy score for the two drugs was calculated according to ZIP model. Error bars: SD (n = 3) [left]. Percentage of growth inhibition after 72h of treatment with the indicated concentrations of IACS-010759 and ibrutinib (IBR) in PDX19. Drug interaction landscape and synergy score for the two drugs was calculated according to ZIP model. Error bars: SD (n = 3) [right]. Note that a ZIP score >10 reflects a synergistic effect, otherwise is an additive effect.

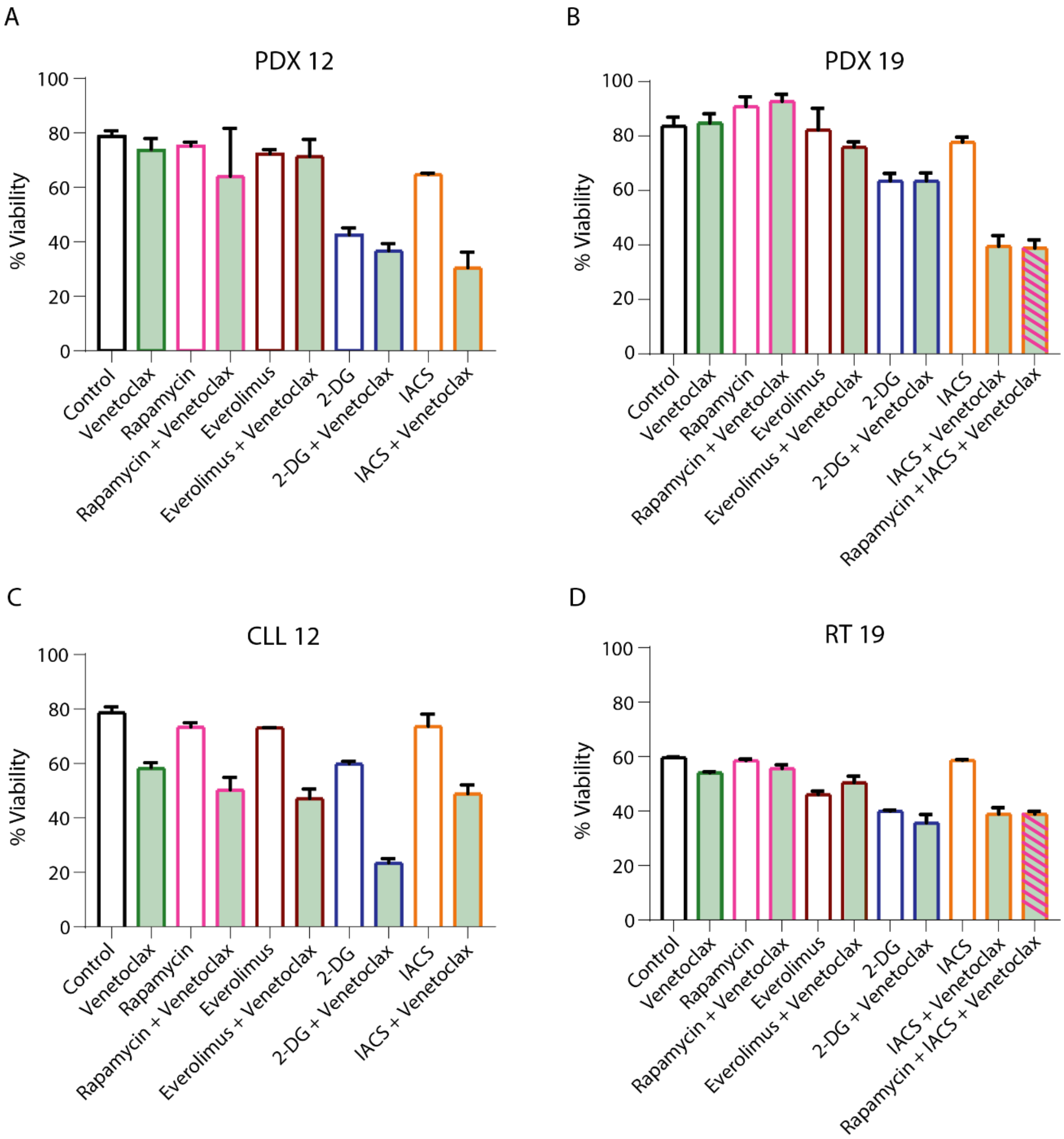

Figure S12. Cytotoxic effect of the pharmacological inhibition of upregulated pathways in PDX samples. Effect on cell viability after 48 hours of treatment with mTOR1 inhibitors (Rapamycin [1μM] and Everolimus [5μM]), glycolysis inhibitor (2-deoxyglucose [2-DG; 5mM]) and OXPHOS inhibitor (IACS [100nM]) with or without venetoclax [75nM] combination. Error bars: SD (n=2).

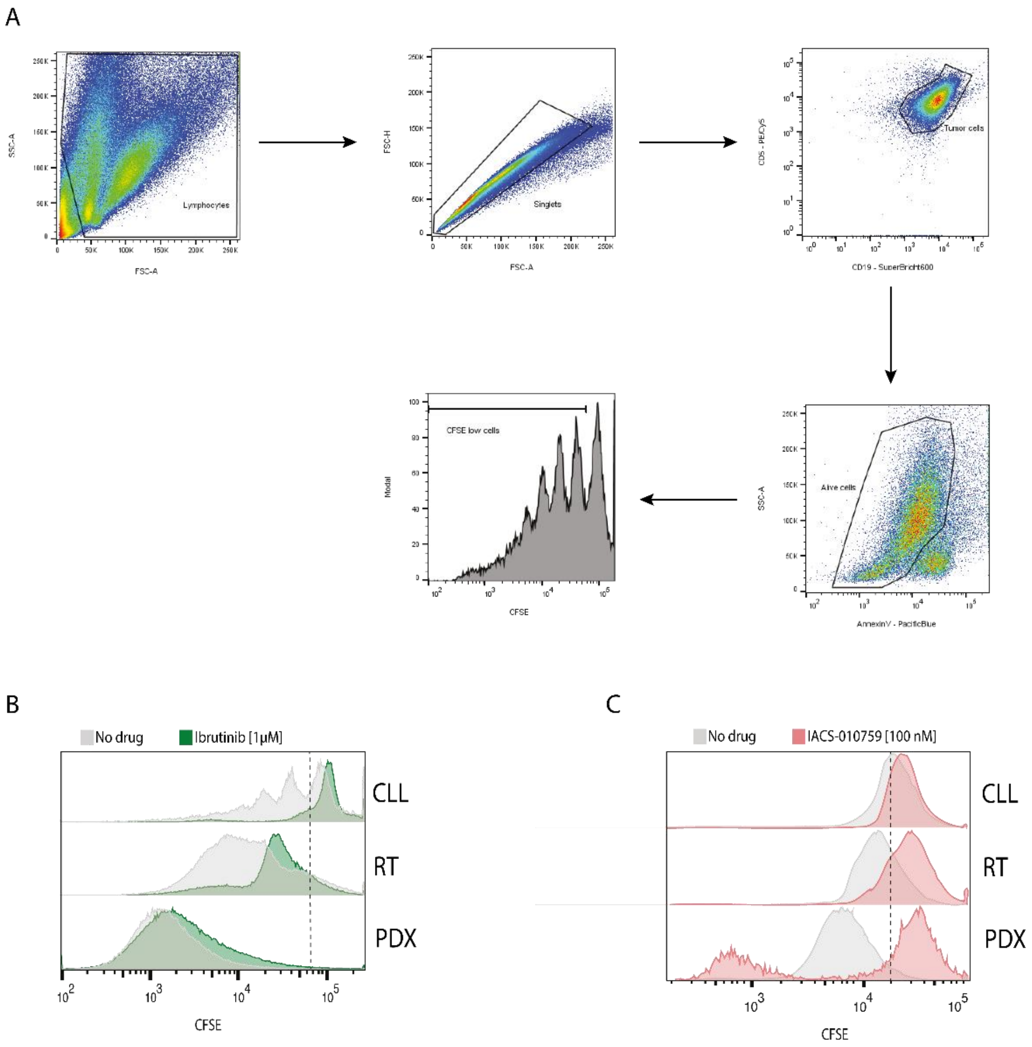

Fig. S13. Flow cytometry gating strategy. A Flow cytometry gating strategy for proliferating cells. Gating analyses were as follow: cell identification in FSC-A vs. SSC-A plot, singlet identification in FSC-A vs. FSC-H plot, tumoral cells ($CD19^{+}$ $CD5^{+}$) in CD19 – (SuperBright600) vs. CD5 – (PE-Cy5) plot, alive cells in AnnexinV – (Pacific Blue) vs. SSC-A plot and proliferating cells in CFSE histogram. **B** CLL, RT and PDX cells treated with ibrutinib to assess growth inhibition. Treated (green) and non-treated (gray) cells with ibrutinib at 1 µM for 6 days are represented in histograms as CFSE intensity vs. normalized cell count. **C** CLL, RT and PDX cells treated with IACS-010759 to assess growth inhibition. Treated (red) or non-treated (gray) cells with IACS-010759 at 100 nM for 72 hours are represented in histograms as CFSE intensity vs. normalized cell count.

Supplemental Tables

Supplemental Tables are placed in the Supplemental Tables Excel file.

Table S1. Clinical and biological characteristics of CLL patients.

Table S2. Flow cytometry antibodies.

Table S3. WGS metadata.

Table S4. Immunoglobulin gene rearrangement and oncogenic translocations.

Table S5. Somatic mutations (WGS, chromosomes 1-22 and X).

Table S6. Somatic coding mutations (WGS, chromosomes 1-22 and X).

Table S7. Copy number alterations.

Table S8. Structural variants.

Table S9. Subclonal reconstruction.

Table S10. MYC amplification in case/PDX19 by FISH.

Table S11. Mutational signatures found in each subclone.

Table S12. RNA-seq metadata.

Table S13. Mean fluorescent ratio Indo-1(violet)/Indo-1(blue).

Table S14. Oxygen consumption measurements in patients' CLL, RT and PDX samples.

Table S15. Summary oxygen consumption in patients' CLL, RT and PDX samples.

Table S16. Flow cytometry gating strategy (Ibrutinib treatment).

Table S17. Flow cytometry gating strategy (Venetoclax treatment).

Table S18. Flow cytometry gating strategy (IACS-010759 treatment).

Table S19. IACS-010759 - Venetoclax combination analysis.

Table S20. IACS-010759 - Ibrutinib combination analysis.

Supplemental References

1. van Dongen JJM, Langerak AW, Brüggemann M, et al. Design and standardization of PCR primers and protocols for detection of clonal immunoglobulin and T-cell receptor gene recombinations in suspect lymphoproliferations: report of the BIOMED-2 Concerted Action BMH4-CT98-3936. *Leukemia*. 2003;17(12):2257–2317.

2. Nadeu F, Royo R, Massoni-Badosa R, et al. Detection of early seeding of Richter transformation in chronic lymphocytic leukemia. *Nat Med*. 2022;28(8):1662–1671.

3. Li H, Durbin R. Fast and accurate short read alignment with Burrows–Wheeler transform. *Bioinformatics*. 2009;25(14):1754–1760.

4. Ahdesmäki MJ, Gray SR, Johnson JH, Lai Z. Disambiguate: An open-source application for disambiguating two species in next generation sequencing data from grafted samples [version 2; peer review: 3 approved]. *F1000Res*. 2016;5:2741.

5. Nadeu F, Mas-de-les-Valls R, Navarro A, et al. IgCaller for reconstructing immunoglobulin gene rearrangements and oncogenic translocations from whole-genome sequencing in lymphoid neoplasms. *Nat Commun*. 2020;11(1):3390–3400.

6. Robinson JT, Thorvaldsdóttir H, Winckler W, et al. Integrative genomics viewer. *Nat Biotechnol*. 2011;29(1):24–26.

7. Jones D, Raine KM, Davies H, et al. cgpCaVEManWrapper: Simple Execution of CaVEMan in Order to Detect Somatic Single Nucleotide Variants in NGS Data. *Curr Protoc Bioinformatics*. 2016;56:15.10.1-15.10.18.

8. McKenna A, Hanna M, Banks E, et al. The Genome Analysis Toolkit: a MapReduce framework for analyzing next-generation DNA sequencing data. *Genome Res*. 2010;20(9):1297–1303.

9. Fan Y, Xi L, Hughes DST, et al. MuSE: accounting for tumor heterogeneity using a sample-specific error model improves sensitivity and specificity in mutation calling from sequencing data. *Genome Biol*. 2016;17(1):178.

10. Danecek P, Bonfield JK, Liddle J, et al. Twelve years of SAMtools and BCFtools. *Gigascience*. 2021;10(2):giab008.

11. Moncunill V, Gonzalez S, Beà S, et al. Comprehensive characterization of complex structural variations in cancer by directly comparing genome sequence reads. *Nat Biotechnol*. 2014;32(11):1106–1112.

12. Raine KM, Hinton J, Butler AP, et al. cgpPindel: Identifying Somatically Acquired Insertion and Deletion Events from Paired End Sequencing. *Curr Protoc Bioinformatics*. 2015;52:15.7.1-15.7.12.

13. Wala JA, Bandopadhayay P, Greenwald NF, et al. SvABA: genome-wide detection of structural variants and indels by local assembly. *Genome Res*. 2018;28(4):581–591.

14. Rimmer A, Phan H, Mathieson I, et al. Integrating mapping-, assembly- and haplotype-based approaches for calling variants in clinical sequencing applications. *Nat Genet*. 2014;46(8):912–918.

15. Cingolani P, Platts A, Wang LL, et al. A program for annotating and predicting the effects of single nucleotide polymorphisms, SnpEff: SNPs in the genome of Drosophila melanogaster strain w1118; iso-2; iso-3. *Fly (Austin)*. 2012;6(2):80–92.

16. Nik-Zainal S, van Loo P, Wedge DC, et al. The life history of 21 breast cancers. *Cell*. 2012;149(5):994–1007.

17. Raine KM, van Loo P, Wedge DC, et al. ascatNgs: Identifying Somatically Acquired Copy-Number Alterations from Whole-Genome Sequencing Data. *Curr Protoc Bioinformatics*. 2016;56:15.9.1-15.9.17.

18. Nik-Zainal S, Davies H, Staaf J, et al. Landscape of somatic mutations in 560 breast cancer whole-genome sequences. *Nature*. 2016;534(7605):47–54.

19. Rausch T, Zichner T, Schlattl A, et al. DELLY: structural variant discovery by integrated paired-end and split-read analysis. *Bioinformatics*. 2012;28(18):i333–i339.

20. Maura F, Bolli N, Angelopoulos N, et al. Genomic landscape and chronological reconstruction of driver events in multiple myeloma. *Nat Commun*. 2019;10(1):3835.

21. Maura F, Degasperi A, Nadeu F, et al. A practical guide for mutational signature analysis in hematological malignancies. *Nat Commun*. 2019;10(1):2969.

22. Alexandrov LB, Kim J, Haradhvala NJ, et al. The repertoire of mutational signatures in human cancer. *Nature*. 2020;578(7793):94–101.

23. Kopylova E, Noé L, Touzet H. SortMeRNA: fast and accurate filtering of ribosomal RNAs in metatranscriptomic data. *Bioinformatics*. 2012;28(24):3211–3217.

24. Bolger AM, Lohse M, Usadel B. Trimmomatic: a flexible trimmer for Illumina sequence data. *Bioinformatics*. 2014;30(15):2114–2120.

25. Bray NL, Pimentel H, Melsted P, Pachter L. Near-optimal probabilistic RNA-seq quantification. *Nat Biotechnol*. 2016;34(5):525–527.

26. Love MI, Huber W, Anders S. Moderated estimation of fold change and dispersion for RNA-seq data with DESeq2. *Genome Biol*. 2014;15(12):550.

27. Hänzelmann S, Castelo R, Guinney J. GSVA: gene set variation analysis for microarray and RNA-seq data. *BMC Bioinformatics*. 2013;14:7.

28. Mongini PKA, Gupta R, Boyle E, et al. TLR-9 and IL-15 Synergy Promotes the In Vitro Clonal Expansion of Chronic Lymphocytic Leukemia B Cells. *J Immunol*. 2015;195(3):901–923.

29. Giménez N, Schulz R, Higashi M, et al. Targeting IRAK4 disrupts inflammatory pathways and delays tumor development in chronic lymphocytic leukemia. *Leukemia*. 2020;34(1):100–114.

30. Garcia-Herrera A, Song JY, Chuang S-S, et al. Nonhepatosplenic γδ T-cell Lymphomas Represent a Spectrum of Aggressive Cytotoxic T-cell Lymphomas With a Mainly Extranodal Presentation. *American Journal of Surgical Pathology*. 2011;35(8):1214–1225.

31. Dühren-von Minden M, Übelhart R, Schneider D, et al. Chronic lymphocytic leukaemia is driven by antigen-independent cell-autonomous signalling. *Nature*. 2012;489(7415):309–312.

32. Lee-Vergés E, Hanna BS, Yazdanparast H, et al. Selective BTK inhibition improves bendamustine therapy response and normalizes immune effector functions in chronic lymphocytic leukemia. *Int J Cancer*. 2019;144(11):2762–2773.
